# Associations between prenatal per- and polyfluoroalkyl substances (PFAS) exposure and fetal growth measurements in utero and at birth in the LIFECODES cohort: 2006–2019^☆^

**DOI:** 10.1016/j.envres.2026.124279

**Published:** 2026-03-15

**Authors:** Ram C. Siwakoti, Danielle Stevens, Kelly K. Ferguson, David E. Cantonwine, Amber Cathey, Bhramar Mukherjee, Thomas F. McElrath, John D. Meeker

**Affiliations:** a Department of Environmental Health Sciences, University of Michigan School of Public Health, Ann Arbor, MI, USA; b Division of Epidemiology, Department of Radiology, University of North Carolina at Chapel Hill, Chapel Hill, NC, USA; c Epidemiology Branch, Division of Intramural Research, National Institute of Environmental Health Sciences, National Institutes of Health, Department of Health and Human Services, Research Triangle Park, NC, USA; d Division of Maternal-Fetal Medicine, Brigham and Women’s Hospital, Harvard Medical School, Boston, MA, USA; e Department of Statistics and Data Science, Yale School of Public Health, New Haven, CT, USA

## Abstract

**Introduction:**

Per- and polyfluoroalkyl substances (PFAS) are a group of synthetic chemicals used in consumer and industrial products, and have been linked with adverse pregnancy outcomes, including altered fetal growth. Few studies, however, have investigated associations between PFAS and longitudinal measures of fetal growth.

**Objective:**

Examine associations between prenatal PFAS and ultrasound measures [femur length (FL), head circumference (HC), abdominal circumference (AC)], and birthweight outcomes in the LIFECODES birth cohort.

**Methods:**

This study included 1220 participants from nested case-control studies within the LIFECODES cohort. We used generalized estimating equations to evaluate associations between six PFAS and repeated fetal growth measurements converted to gestational age (GA)-specific Z-scores, while multivariable linear and logistic regression models assessed associations with birthweight and size-for-GA [small-for-GA (SGA) and large-for-GA (LGA)], respectively. The effect of PFAS mixture was examined using quantile-based g-computation (qgcomp). All models were adjusted for maternal age, race/ethnicity, educational attainment, health insurance status, pre-pregnancy body mass index, parity, enrollment year, fetal sex, and aspects of study design.

**Results:**

An interquartile range increase in perfluorooctanoic acid (PFOA) was associated with a 0.10 standard deviation (SD) increase in FL Z-score (95% CI: 0.02, 0.18). Positive associations with FL were more pronounced among African American participants [SD change: 0.26 (95% CI: −0.01, 0.53)] and male fetuses [SD change: 0.16 (95% CI: 0.05, 0.28)]. Qgcomp analyses showed similar positive associations with FL. Associations with birthweight outcomes were generally inconsistent, with certain PFAS associated with increased odds of LGA among females, while some PFAS were linked to lower odds of SGA across sexes.

**Conclusions:**

Prenatal PFAS exposure was modestly associated with increased FL, with some variations by fetal sex or maternal race. Future research should examine underlying biological mechanisms, clinical implications, and the influence of modifying factors such as psychosocial stress and maternal diet on the observed associations.

## 1. Introduction

Altered fetal growth, characterized by deviations from the expected growth trajectory, is an important concern for the health of a newborn, both during and after birth (Damhuis et al., 2021). These deviations, most often presenting as intrauterine growth restriction (IUGR) or as macrosomia — indicating restricted (Suhag and Berghella, 2013) or excessive growth (Boulet et al., 2003), respectively — are associated with an increased risk of adverse outcomes, such as preterm delivery and neonatal morbidity or mortality (Jolly et al., 2003; Chernausek, 2012; Henriksen, 2008; Colella et al., 2018; Bamberg et al., 2013). Beyond the perinatal period, affected infants may also face a greater likelihood of long-term health challenges such as neurodevelopmental impairment, cardiovascular disease, and metabolic syndrome (Jolly et al., 2003; Chernausek, 2012; Henriksen, 2008; Colella et al., 2018; Bamberg et al., 2013; Gluckman et al., 2008). Fetal growth abnormalities occur along a continuum and arise from a diverse array of risk factors, most commonly maternal malnutrition and medical conditions, fetal genetic anomalies or infections, and placental dysfunction (Frigoletto and Rothchild, 1977; Ounsted et al., 1981; Langer, 2000). In addition, maternal smoking (Andres and Day, 2000) and exposure to various environmental toxicants (Street and Bernasconi, 2020; Kamai et al., 2019) have been shown to further elevate these risks. Growing evidence, including findings from our group, also implicate environmental contaminants, such as per- and polyfluoroalkyl substances (PFAS), in influencing fetal growth and development, and other adverse pregnancy outcomes (Säve-Söderbergh et al., 2024; Gao et al., 2021; Bach et al., 2015; Siwakoti et al., 2023a).

PFAS are a large class of synthetic chemicals that have been frequently used in industrial applications and consumer products, owing to their unique physicochemical properties, specifically resistance to heat, water, and grease (Buck et al., 2011; Calafat et al., 2006). Their chemical stability, along with their widespread use in products such as nonstick cookware, stain or water-resistant fabrics, firefighting foams, and cleaning agents, has led to their persistence in the environment and bioaccumulation in living organisms worldwide (Buck et al., 2011; Sunderland et al., 2019). Despite the phase-out of certain legacy PFAS due to health concerns (USEPA: PFAS National Primary Drinking, 2023), these chemicals continue to be detected in the blood of over 90% of Americans (Kato et al., 2018; CDC, 2024), with primary sources of exposure in the general population being drinking water, contaminated food, and household dust (Sunderland et al., 2019). As a result, there are growing concerns related to the health effects of PFAS, especially for pregnant women and developing fetuses (Sunderland et al., 2019). Several epidemiological studies have linked elevated prenatal PFAS exposure with a range of adverse pregnancy outcomes like preterm birth, preeclampsia, or alteration of birthweight across different populations (Gao et al., 2021; Padula et al., 2023; Bommarito et al., 2021).

Recently, increased emphasis has been placed on examining the effects of prenatal environmental exposures on ultrasound parameters of fetal growth (Bommarito et al., 2023a, 2023b, 2024a, 2024b). These measurements, such as head circumference (HC), abdominal circumference (AC), femur length (FL), and estimated fetal weight (EFW), provide a dynamic picture of fetal growth and development in utero (Debbink et al., 2020). Specifically, the use of repeated ultrasound measurements throughout pregnancy offers a unique opportunity to capture longitudinal changes in fetal growth that may not be apparent when relying solely on a single time-point assessment such as birthweight. However, only a handful of epidemiological studies so far have examined PFAS in relation to these measurements, with inconsistent findings (Ouidir et al., 2020; Peterson et al., 2022; Yang et al., 2025; Costa et al., 2019; Knox et al., 2024). For example, while some studies, including that by Ouidir et al. (2020) have reported positive associations between prenatal maternal PFAS levels and FL, others have found null (Peterson et al., 2022; Costa et al., 2019; Knox et al., 2024) or inverse (Peterson et al., 2022; Yang et al., 2025; Costa et al., 2019) associations that varied by PFAS compound, maternal behaviors (e.g., smoking, psychosocial stress), or fetal sex. Most prior studies have been limited by relatively modest sample sizes (N < 750 in three of the five published studies) and by homogeneous study populations, which restricts generalizability and reduces statistical power, highlighting the need for additional research on this topic.

The current study aimed to address these gaps by evaluating associations between prenatal PFAS exposure and repeated ultrasound parameters of fetal growth, as well as birthweight outcomes, in a large, racially and socioeconomically diverse U.S. pregnancy cohort. Furthermore, we explored whether the effects of PFAS on fetal growth are modified by fetal sex and whether these associations differ across categories of maternal race.

## 2. Methods

### 2.1. Study population

The current study includes a subset of participants from the LIFECODES study, a prospective birth cohort established at Brigham and Women’s Hospital (BWH) in Boston, MA. The details on the LIFECODES study design and participant selection have been published previously (Ferguson et al., 2014; Cantonwine et al., 2015). Briefly, participants were eligible for enrollment into the LIFECODES study if they initiated prenatal care before 15 weeks of gestation, were at least 18 years of age, and planned to deliver at BWH. Higher-order multiple gestations (triplets or more) were excluded. Participants provided plasma samples at four timepoints as part of the broader LIFECODES protocol: the first study visit [median gestational age (GA) = 10.9 weeks], and approximately 18, 26, and 35 weeks of gestation. In the current study, PFAS were quantified only from samples collected at the first visit, and in some instances, second visit (see PFAS measurement section). GA was determined using the last menstrual period and ultrasound measurements following American College of Obstetricians and Gynecologists guidelines (ACOG: Committee Opinion No 700, 2017).

For this analysis, we considered 1311 women who had plasma samples available for PFAS measurements. Among them, 469 (127 preterm cases and 342 controls) were selected as part of a nested case-control study of preterm birth conducted between 2006 and 2008 (Ferguson et al., 2014; Cantonwine et al., 2015). We also included an additional 720 participants (250 preterm cases and 470 controls) from a more recent nested case-control study of preterm birth. Controls were randomly selected from participants delivering at or after 37 weeks. To maximize our sample size, we further included 122 participants that were part of pilot analyses examining the effects of PFAS on fetal growth outcomes (N = 83) or preeclampsia (N = 39). From this pool, our final analytic sample comprised 1220 women (355 preterm cases and 865 controls) who had at least one ultrasound scan between 16 and 41 weeks of gestation. Due to the increased likelihood of selection of participants with preterm delivery, we applied sampling weights for cases and controls in statistical modeling, and ran sensitivity analyses limited to only case-control participants (see Sensitivity analysis section for details). All study protocols received approval from the Institutional Review Boards at the BWH and the University of Michigan, and all participants provided written informed consent prior to enrollment.

### 2.2. PFAS measurement

We quantified nine PFAS in maternal plasma samples collected in early pregnancy (median GA = 10.9 weeks). The targeted compounds included N-methylperfluoro-1-octanesulfonamidoacetic acid (MPAH), perfluorodecanoic acid (PFDA), perfluoroheptanoic acid (PFHP), perfluorohexane sulfonic acid (PFHxS), perfluorononanoic acid (PFNA), perfluorooctanoic acid (PFOA), perfluorooctane sulfonic acid (PFOS), perfluorooctane sulfonamide (PFOSA), and perfluoroundecanoic acid (PFUA). These specific PFAS were selected based on their prevalence in the U.S. population during the study period and the availability of reliable laboratory standards for quantification (Calafat et al., 2019).

Analyses were conducted at NSF International (Ann Arbor) using a validated protocol based on the Centers for Disease Control and Prevention’s Polyfluoroalkyl Chemicals Method No. 6304.1, the details of which are described elsewhere (Kato et al., 2009; Siwakoti et al., 2024). Briefly, plasma samples were stored at −80 °C, thawed, and vortexed to ensure homogeneity. Method blanks, calibration standards, quality-control (QC) samples, and isotopically labeled internal standards were prepared according to standardized protocols, followed by on-line solid-phase extraction (SPE) for preconcentration. Subsequent analyses were performed using liquid chromatography–tandem mass spectrometry (LC-MS/MS) in negative-ion mode, with instrument calibration checks consistently achieving a correlation coefficient (R^2^) ≥ 0.998, 90–108% accuracy, and relative standard deviations (RSD) of 1.6–8%. The limit of detection (LOD) was 0.5 ng/mL for PFOA and 0.1 ng/mL for all other PFAS.

Five PFAS, namely PFOS, PFOA, PFNA, PFHxS, and PFDA were detected in more than 80% of samples, while PFUA and MPAH were detected in 68.2% and 45.3% of samples, respectively. For this study, we included six PFAS, including PFUA, in subsequent modeling, while MPAH (45.33%), PFHP (18.85% detection), and PFOSA (0.33% detection) were excluded from further consideration. For sample concentrations below the LOD, machine-read values were used if available; otherwise, the values were imputed using LOD/√2. Because distributions of PFAS concentrations were right-skewed, we natural log-transformed them to minimize the influence of outliers in subsequent analyses.

### 2.3. Assessment of ultrasound parameters of fetal growth

Detailed information on ultrasound measurements in LIFECODES has been published elsewhere (Bommarito et al., 2023a, 2024a). In summary, we abstracted ultrasound measurements (mm) of HC, AC, and FL from participant medical records. Using these measurements, we derived EFW (g), based on Hadlock’s formula #3, which incorporates HC, AC, and FL as shown below (Hadlock et al., 1985):

$${\text{log}}_{10}\left(\text{EFW}\right)=1.326-0.00326\times AC\times FL+0.0107\times HC+0.0438\times AC+0.158\times FL$$

For 30 ultrasound records from 27 participants where HC data were unavailable, we applied an alternative Hadlock formula based on biparietal diameter (BPD):

$${\text{log}}_{10}(\text{EFW})=1.335-0.0034\times AC\times FL+0.0316\times BPD+0.0457\times AC+0.1623\times FL$$

We considered all scans taken between 16 and 41 weeks of gestation, and excluded those before 16 weeks due to limited between-subject variability in early ultrasound fetal growth measurements and the absence of robust internal standards for this period. We did not investigate BPD as a standalone parameter because it is highly correlated with HC (Pearson’s r = 0.84 in the current study), which is generally preferred over BPD as a measure of fetal head size given that it is less influenced by head shape (Loughna et al., 2009).

All measurements were converted into GA-specific Z-scores using an internal reference standard developed from the BWH obstetric population (Cantonwine et al., 2016). For participants with multiple scans in the same gestational week, we averaged those measurements for analysis (applies to ~ 0.64% of scans) since we could not reliably distinguish the clinical rationale for repeat scans within the same week. We identified and manually verified extreme values (exceeding four SDs from zero): 1 record for HC, 3 for AC, 6 for FL, and 1 for EFW. Given their small number, these outliers were retained in the final analytic dataset (5016 records for HC, 5046 for AC, 5188 for FL, and 5039 for EFW), with a sensitivity analysis conducted excluding these outliers.

### 2.4. Assessment of birthweight measurements

We abstracted GA (weeks) at delivery and birthweight (g) from medical records. All birthweights were converted to a GA-specific Z-score (birthweight Z-score) using similar internal growth standards applied to ultrasound measurements (Cantonwine et al., 2016). We also considered two categorical measures of extreme birthweight commonly used in clinical and epidemiological research: small-for-GA (SGA), defined as a birthweight Z-score at or below the 10th percentile, and large-for-GA (LGA), defined as a birthweight Z-score at or above the 90th percentile.

### 2.5. Covariate assessment

We collected data on maternal demographics, socioeconomic characteristics, and medical history through self-administered questionnaires at the initial visit. Baseline covariates included maternal age (in years), race/ethnicity (categorized as non-Hispanic White, African American, Hispanic, Asian, Mixed, or Other), pre-pregnancy body mass index (BMI) calculated from self-reported weight and height, health insurance status (private/HMO or Medicaid/uninsured), educational attainment (high school or less, some college, or undergraduate degree or higher), nulliparity (yes/no), and smoking or alcohol use during pregnancy (yes/no for both). We also recorded the year of PFAS sample collection based on enrollment date and documented fetal sex at delivery. Given the small number of participants identifying as Mixed or Other race, we combined these participants in a single category for the purpose of statistical analysis. Race is a social construct and its inclusion in our analysis is meant to account for structural inequities and other unmeasured factors that may influence both exposure and health outcomes (Williams et al., 2016).

We identified potential confounders based on previous literature (Ouidir et al., 2020; Peterson et al., 2022; Yang et al., 2025; Costa et al., 2019; McAdam and Bell, 2023) and through a directed acyclic graph (DAG) (Fig. S1). All primary models were adjusted for maternal age, race, educational attainment, insurance status, pre-pregnancy BMI, and nulliparity status. We further controlled for enrollment year to capture temporal trends in PFAS exposure, and fetal sex to account for sex-specific differences in growth measurements. Additionally, because participants with more frequent ultrasound monitoring may differ systematically from those with fewer scans (e.g., due to clinical complications), we adjusted ultrasound models for the total number of scans per participant, where multiple scans within the same gestational week were counted only once.

### 2.6. Descriptive statistics

We summarized demographic and socioeconomic characteristics of study participants by reporting medians and interquartile ranges [IQRs; defined by the 25th (Q1) and 75th (Q3) percentiles] for continuous variables, and frequencies and proportions for categorical variables. The distributions of PFAS among a smaller subset of LIFECODES participants have been previously published (Bommarito et al., 2021; Siwakoti et al., 2023b). We reevaluated these distributions in a larger sample by reporting detection rates (%) and medians (Q1, Q3) among all participants, as well as stratified by maternal and fetal characteristics. We also examined fetal growth measurements during pregnancy and at birth across different maternal demographic categories. Finally, we calculated Spearman correlation coefficients (*ρ*) (Myers and Sirois, 2004) among different PFAS, and among different fetal growth measurements to assess their interrelatedness.

### 2.7. Pairwise associations between PFAS and fetal growth measures

We used generalized estimating equation (GEE) models (‘geepack’ package in R) with an exchangeable correlation structure and Gaussian family to estimate covariate-adjusted associations between each PFAS and Z-scores of repeated ultrasound parameters of fetal growth (Højsgaard et al., 2006). The beta coefficients for PFAS from these models, representing changes in Z-score per unit increase in log-transformed PFAS concentration, were expressed as changes in Z-score per IQR increase in PFAS levels. We examined birthweight Z-score using separate multivariable linear regression models, adjusting for the same set of covariates (except the number of scans per participant) used in the analysis of the repeated ultrasound parameters. For extreme birthweight categories (SGA and LGA), we used multivariable logistic regression models, treating appropriate-for-GA (AGA, 10th-90th percentile of birthweight Z-score) as the reference category. The effects in these models were expressed as odds ratios (ORs) per IQR increase in levels of each PFAS.

We conducted two secondary analyses to explore potential effect modifiers. First, to examine whether the associations between PFAS and fetal growth differed by fetal sex, we tested for effect modification by including a product interaction term between each PFAS and fetal sex in our models, then derived fetal sex-specific estimates. Second, we investigated whether the associations varied across maternal racial/ethnic groups by obtaining race/ethnicity-specific estimates for each PFAS-outcome pair.

We applied sampling weights to account for the selection of preterm cases and controls (weight for preterm cases = 1.40; weight for controls = 4.12) in our study. These weights were calculated by comparing the total number of cases and controls with available PFAS data to the total number of participants with similar demographic characteristics available for selection. We conducted all primary analyses using complete-case datasets (N = 4825, 4854, and 4847 records from 1126 participants for HC, AC, and EFW, respectively; N = 4980 records from 1173 participants for FL), excluding participants with missing primary covariates (see Table 1 for details on missingness). We conducted all hypothesis testing at *α* = 0.05. However, based on the recommendations from the authoritative scientific bodies, we interpreted our findings based on the magnitude, direction, and variability of our estimates rather than the p-values alone (Savitz et al., 2024).

### 2.8. Associations between PFAS mixture and fetal growth measures

In addition to the pairwise single-pollutant analysis, we also examined associations between a PFAS mixture and fetal growth outcomes to evaluate the joint effect of PFAS that are correlated (Calafat et al., 2007) and co-occur (USEPA: PFAS National Primary Drinking, 2023) in human populations. We modeled the mixture of PFOA, PFOS, PFNA, PFHxS, PFDA, and PFUA using quantile-based g-computation (qgcomp) (Keil et al., 2020, 2021). This method extends parametric g-computation to estimate the overall effect of simultaneously increasing all exposures by one quantile, modeling the joint effect as a weighted linear index of quantized exposures. For repeated ultrasound measurements, we implemented the bootstrapped version of qgcomp (using the function ‘qgcomp.glm.boot’ in R) with participant-level resampling so that all measurements from the same participant were sampled together. Code for this analysis is available at: https://github.com/ramsiwakoti/PFA S-Ultrasound-QGcomp. For birthweight Z-score, we applied a standard (non-bootstrapped) implementation of qgcomp. The primary parameter estimate (ψ_1_) for continuous outcomes represented the expected change in Z-score when concentrations of all PFAS were simultaneously increased by one quantile. All models included the same set of covariates or sampling weights used in the pairwise analysis.

### 2.9. Exploratory ultrasound fetal growth trajectory modeling

We conducted an ad hoc exploratory analysis using multivariate latent class trajectory modeling (LCTM) to identify subgroups of fetuses with distinct longitudinal patterns of AC, FL, and HC Z-scores. LCTM identifies latent classes within a population, each characterized by a distinct average trajectory across multiple ultrasound parameters, capturing developmental heterogeneity that single-time-point measures such as birthweight may miss (Andruff et al., 2009; Arrandale et al., 2006; Nagin and Odgers, 2010). Models were fitted using the ‘PROC TRAJ’ procedure in SAS, specifying GA as the continuous time metric, which allows the model to accommodate variable timing of ultrasound measurements across participants (Arrandale et al., 2006). We also incorporated sampling weights and the number of scans per participant in the individual models. We considered models with three to five classes, comparing Akaike Information Criterion (AIC), Bayesian Information Criterion (BIC), average posterior probabilities of class membership (≥0.75), relative entropy (≥0.75), and minimum class size (≥5%). Relative entropy, calculated using the ‘LCTMtoolkit’ package in R, was used as a measure of class separation quality (Lennon et al., 2018), with higher values indicating better differentiation among classes. We also drew on expert knowledge of fetal growth patterns to guide model selection when more than one solution met minimum criteria. For the selected model, we further refined the specification of polynomial orders for each trajectory, retaining higher-order terms only if their coefficients were statistically significant at *α* = 0.05, unless only a linear term remained.

After selecting the optimal model, participants were assigned to the class with the highest posterior probability of membership. We then summarized birth and clinical characteristics within each class to assess whether distinct growth trajectories corresponded to clinical risks such as preterm delivery or neonatal intensive care unit (NICU) admission. Finally, we examined associations between PFAS concentrations and trajectory class membership using multinomial logistic regression, estimating the relative risk ratio (RRR) of belonging to each class (relative to a reference class) per IQR increase in PFAS, adjusting for the same set of covariates used in the primary analyses.

### 2.10. Sensitivity analysis

We conducted several sensitivity analyses to assess the robustness of our findings. First, to facilitate comparison with previous literature, we reanalyzed ultrasound measurements using raw values instead of Z-scores, adjusting for GA at ultrasound as a nonlinear spline term alongside the primary covariates (Yang et al., 2025). For this, we used the ‘bs’ function in the ‘splines’ package in R with default settings, which correspond to a cubic polynomial basis (degree = 3, no internal knots, boundary knots at the observed range) (Perperoglou et al., 2019). Additionally, we fit linear mixed-effects models with a random intercept to estimate pairwise associations between each PFAS and ultrasound parameters, for comparability with prior studies. Second, we analyzed raw birthweight in addition to birthweight Z-score to accommodate various reporting preferences in regulatory assessments and meta-analyses. While running models for raw birthweight, we similarly adjusted for GA at delivery in addition to the primary covariates. Third, we repeated our primary analysis after excluding the small number of participants who reported smoking during pregnancy. Fourth, to account for hemodynamic changes potentially influencing plasma PFAS concentrations during pregnancy, we adjusted for GA at the time of sample collection used to measure PFAS (Sagiv et al., 2017). Fifth, we conducted analyses excluding extreme fetal growth measurements (| Z-score| > 4) to ensure that our results were not disproportionately influenced by outliers. Finally, to ensure that the effect estimates from our analysis were not overly influenced by the inclusion of data from smaller pilot analyses, we also restricted our primary analysis to participants selected as part of the case-control sample selection only.

## 3. Results

### 3.1. Descriptive statistics

The baseline demographic, behavioral, and clinical characteristics of study participants are included in Table 1. The median maternal age and pre-pregnancy BMI were 32.8 years and 25.0 kg/m^2^, respectively. The majority of participants were non-Hispanic White (56.8%), followed by Hispanic (16.2%), African American (14.7%), and Asian (6.9%). Most participants had at least a bachelor’s degree (67.0%), and the majority were privately insured (73.3%). Approximately 43.0% of participants were nulliparous, and a small proportion of participants reported smoking (6.8%) or drinking alcohol (6.7%) during pregnancy. There was a slightly larger number of male fetuses (53.2%) as compared to female fetuses (46.5%), and about 80.5% of newborns were classified as AGA, while 9.5% were SGA, and 9.3% were LGA. The number of ultrasound scans per participant ranged from 1 to 13, with 89% of participants undergoing at least two scans.

The distributions of PFAS across the entire study period and by enrollment year are summarized in Table 2 and S1. Among the six PFAS detected in more than 68% of participants, PFOS had the highest concentrations, with a median (Q1, Q3) of 4.66 (2.86, 7.45) ng/mL (Table 2). The concentrations of all PFAS showed a declining trend over time, with PFOS [median (Q1, Q3)] decreasing from 7.15 (5.01, 10.00) ng/mL in 2006–2008 to 2.63 (1.71, 3.69) ng/mL in 2015–2019, and PFOA falling from 2.48 (1.76, 3.26) ng/mL to 1.00 (0.65, 1.55) ng/mL over the same period (Fig. S2). Fig. S3 shows the correlations between PFAS compounds, with Spearman’s *ρ* ranging from 0.29 (PFUA and PFHxS) to 0.83 (PFNA and PFDA). Table S1 also presents the unadjusted concentrations of PFAS across different categories of maternal demographic and pregnancy characteristics. In general, participants with higher socioeconomic status, as indicated by private insurance and higher educational attainment, had higher levels of most PFAS. Asian and non-Hispanic White participants had higher PFAS concentrations compared to Black or Hispanic participants. PFAS levels were also consistently higher in nulliparous participants compared to their parous counterparts.

The growth trajectories based on raw measurements of FL, HC, AC, and EFW are visualized in Fig. S4. The correlations between different fetal growth measures are shown in Fig. S5. Among the ultrasound measures, correlations were moderate [Spearman’s *ρ* = 0.57 (HC and FL), 0.53 (HC and AC), and 0.46 (AC and FL)]. All three measures were highly correlated with EFW (Spearman’s *ρ* = 0.65–0.82), and moderately correlated with birthweight (0.36 for FL to 0.58 for AC). Table S2 presents distributions of ultrasound parameters across maternal demographic and clinical characteristics. Generally, fetal growth Z-scores were higher among participants who identified as non-Hispanic White, had BMI above 30, attained higher education, held private insurance, were parous, abstained from alcohol during pregnancy, and carried male fetuses. We did not observe a consistent temporal trend in fetal growth Z-scores across years of sample collection, although there were some year-to-year fluctuations (Table S2). Among all 1,220 participants, the mean GA at the first scan was approximately 17.9 weeks, and among the 1,086 participants who had a second scan, the mean GA was approximately 24.6 weeks (Table S3).

### 3.2. Pairwise associations between PFAS and ultrasound parameters of fetal growth

**Fig. 1.**
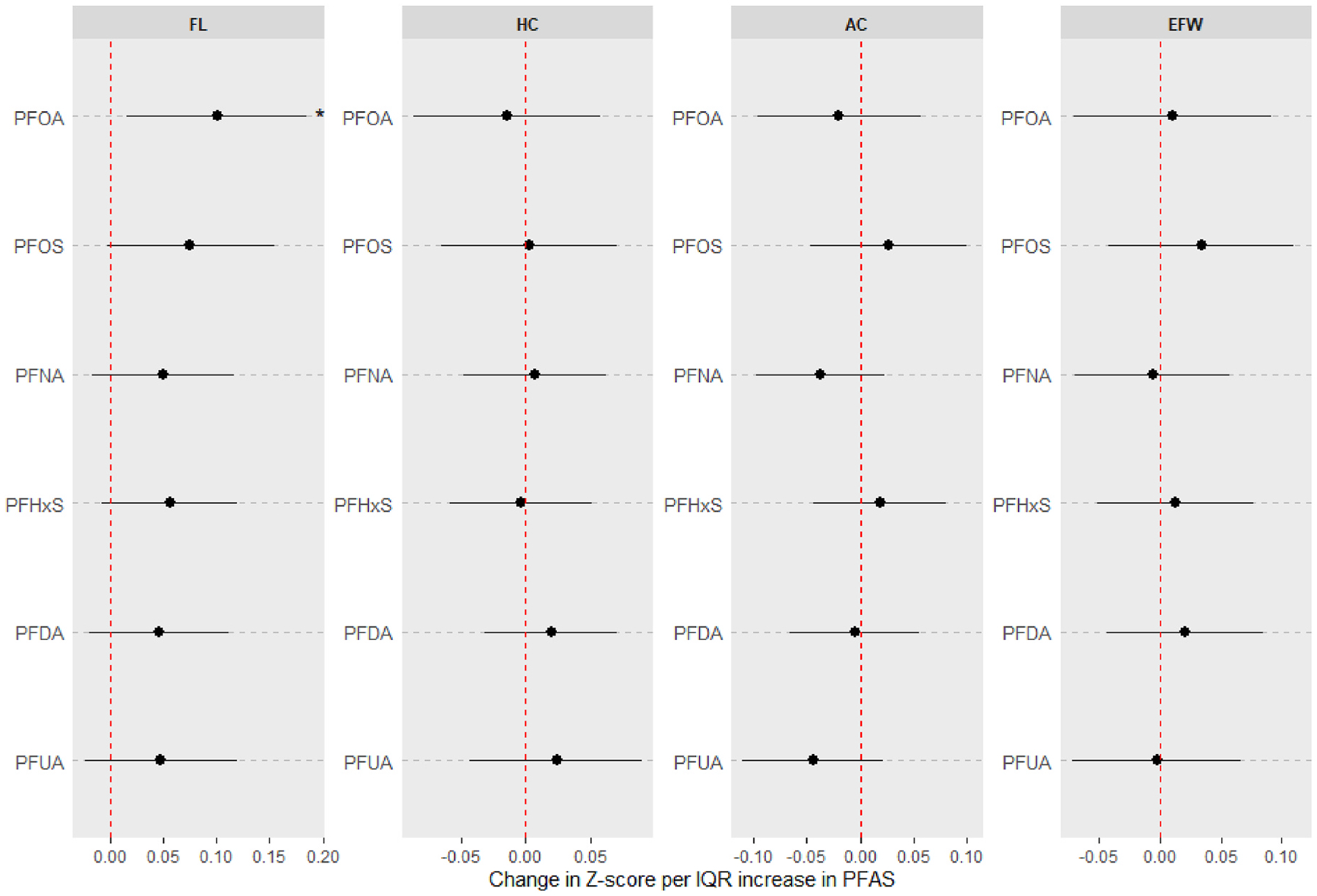
Associations between prenatal PFAS exposure and standardized ultrasound parameters of fetal growth [femur length (FL), head circumference (HC), abdominal circumference (AC), and estimated fetal weight (EFW)]. Estimates derived from generalized estimating equations with exchangeable correlation. Effect estimates (95% confidence intervals) show the change in Z-score (or standard deviation) of each measure per interquartile range increase in log-transformed PFAS concentrations. All models were adjusted for maternal age, prepregnancy BMI, race, education, insurance status, parity, enrollment year, fetal sex and number of ultrasound scans. Asterisks indicate estimates with 95% confidence intervals excluding the null.

Fig. 1 and Table S4 present results from adjusted pairwise associations between individual PFAS compounds and ultrasound parameters of fetal growth Z-scores. We observed a general positive trend in associations between most PFAS and FL. For instance, an IQR increase in PFOA led to a 0.10 (95% CI: 0.02, 0.18) SD increase in FL Z-score. Similarly, IQR increases in PFOS and PFHxS were associated with 0.08 (95% CI: 0, 0.15) and 0.06 (95% CI: −0.01, 0.12) SD increases in FL Z-score, respectively.

**Fig. 2.**
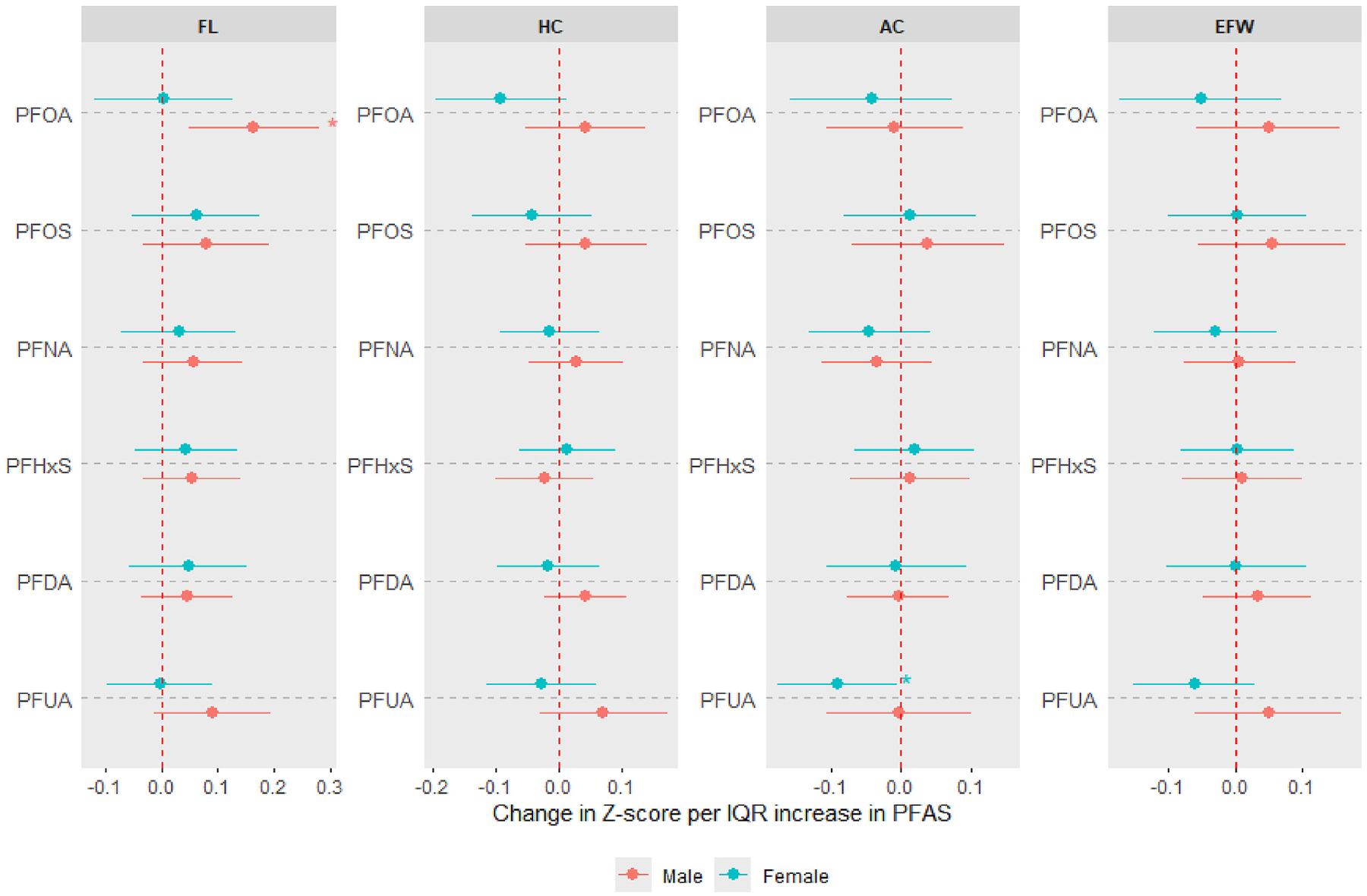
Associations between prenatal PFAS exposure and standardized ultrasound parameters of fetal growth [femur length (FL), head circumference (HC), abdominal circumference (AC) and estimated fetal weight (EFW)], stratified by fetal sex. Estimates derived from generalized estimating equations with exchangeable correlation. Effect estimates (95% confidence intervals) show the change in Z-score (or standard deviation) of each measure per interquartile range increase in log-transformed PFAS concentrations. Separate models were run for each sex. All models were adjusted for maternal age, prepregnancy BMI, race, education, insurance status, parity, enrollment year, and number of ultrasound scans. Asterisks indicate estimates with 95% confidence intervals excluding the null.

In fetal sex-stratified analyses (Fig. 2, Table S4), the positive trend in associations between most PFAS (PFOS, PFNA, PFHxS, PFDA) and FL was generally evident in both sexes, although most 95% confidence intervals included the null. However, the associations of PFUA and PFOA with FL were positive or suggestively positive in male fetuses, but null in female fetuses, with the association between PFUA and AC being inverse in female fetuses and null in males. The p-values corresponding to PFAS x fetal sex interaction terms are provided in Table S5.

**Fig. 3.**
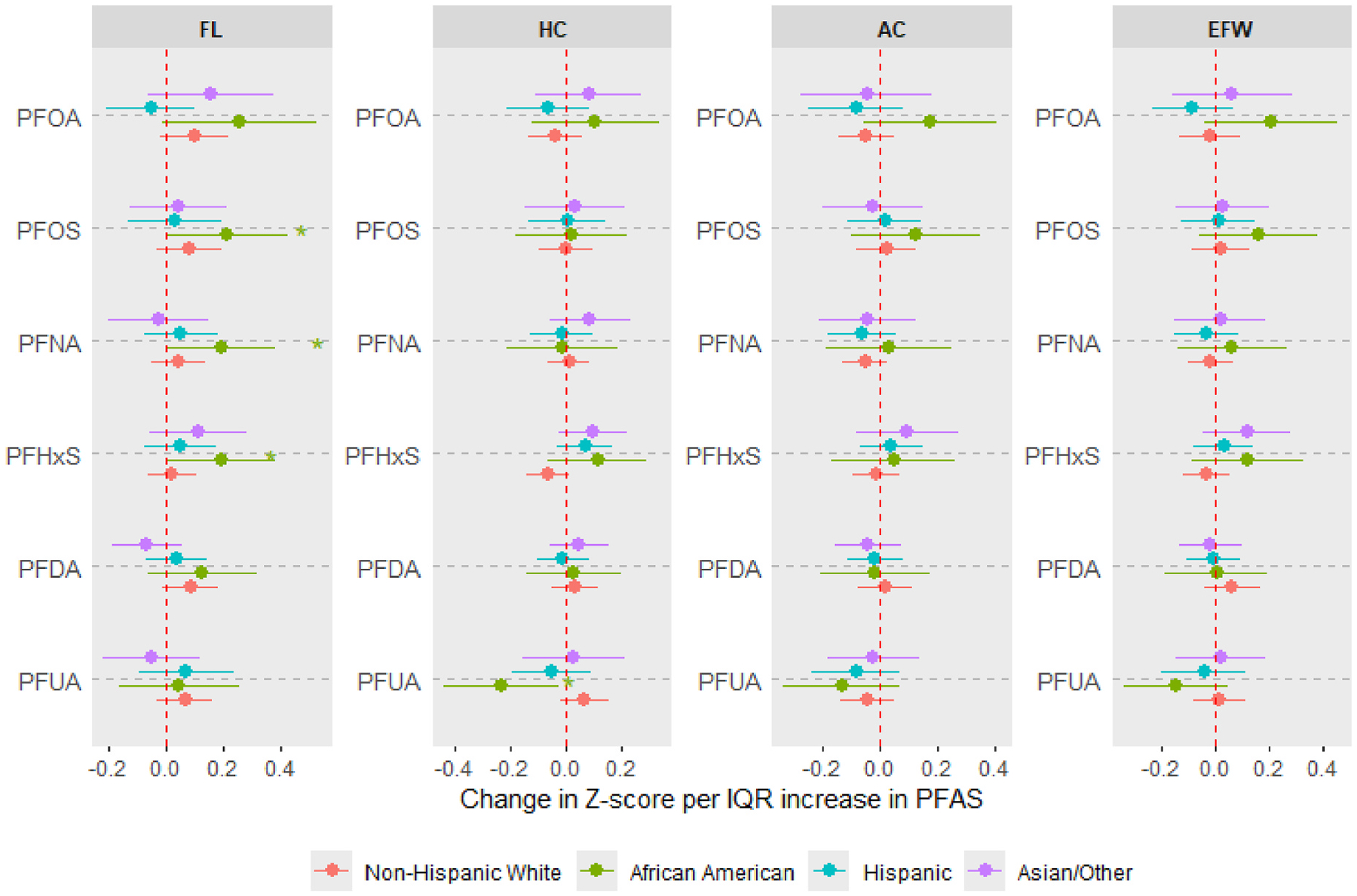
Associations between prenatal PFAS exposure and standardized ultrasound parameters of fetal growth [femur length (FL), head circumference (HC), abdominal circumference (AC) and estimated fetal weight (EFW)], stratified by maternal race. Estimates derived from generalized estimating equations with exchangeable correlation. Effect estimates (95% confidence intervals) show the change in Z-score (or standard deviation) of each measure per interquartile range increase in log-transformed PFAS concentrations. Separate models were run for each racial category. All models were adjusted for maternal age, prepregnancy BMI, education, insurance status, parity, fetal sex, enrollment year, and number of ultrasound scans. Asterisks indicate estimates with 95% confidence intervals excluding the null.

The maternal race-stratified analyses (Fig. 3, Table S6) showed that the positive associations of PFOA and PFOS with FL, AC, and EFW were consistently more pronounced among African American participants compared to participants from other racial groups. Similarly, the positive associations of PFNA and PFHxS with FL were also stronger in African American participants, while the associations between PFUA and HC or EFW were inverse or suggestively inverse. Interestingly, the association between PFHxS and HC was suggestively inverse among non-Hispanic White participants in contrast to positive trends for others.

### 3.3. Pairwise associations between PFAS and measures of fetal growth at birth

The associations between prenatal PFAS and birthweight Z-score were generally null in the overall cohort (Table S7). In fetal sex-stratified analysis, we observed generally positive, but non-significant associations between different PFAS and birthweight Z-score in females, with an IQR increase in PFDA leading to a 0.15 (95% CI: 0.03, 0.27) SD increase in birthweight Z-score in this group. In maternal race-stratified analysis, we observed positive trends in African American participants, with an IQR increase in PFOA associated with a 0.44 (95% CI: 0.15, 0.73) SD increase, and additional suggestive positive associations for PFOS, PFNA, and PFHxS. In contrast, we observed generally inverse trends in Hispanic participants, with the association for PFOA being suggestively significant. Similarly, PFNA showed a suggestively positive association with birthweight Z-score among Asian/Other participants.

Table S8 shows the associations between PFAS and SGA and LGA birth. In the overall cohort, higher levels of PFOS and PFNA were linked to lower odds of LGA, with 18% [OR: 0.82 (95% CI: 0.67, 0.99)] and 15% [OR: 0.85 (95% CI: 0.72, 0.99)] reductions, respectively. Fetal sex-stratified analysis indicated increased odds of LGA for females and decreased odds for males. Additionally, PFOS [OR: 0.72 (95% CI: 0.61, 0.86)] and PFHxS [OR: 0.75 (95% CI: 0.65, 0.88)] were inversely associated with SGA in the overall cohort, with results consistent across sexes. An inverse association between PFDA and SGA was also observed among females.

### 3.4. Associations between PFAS mixture and measures of fetal growth

**Fig. 4.**
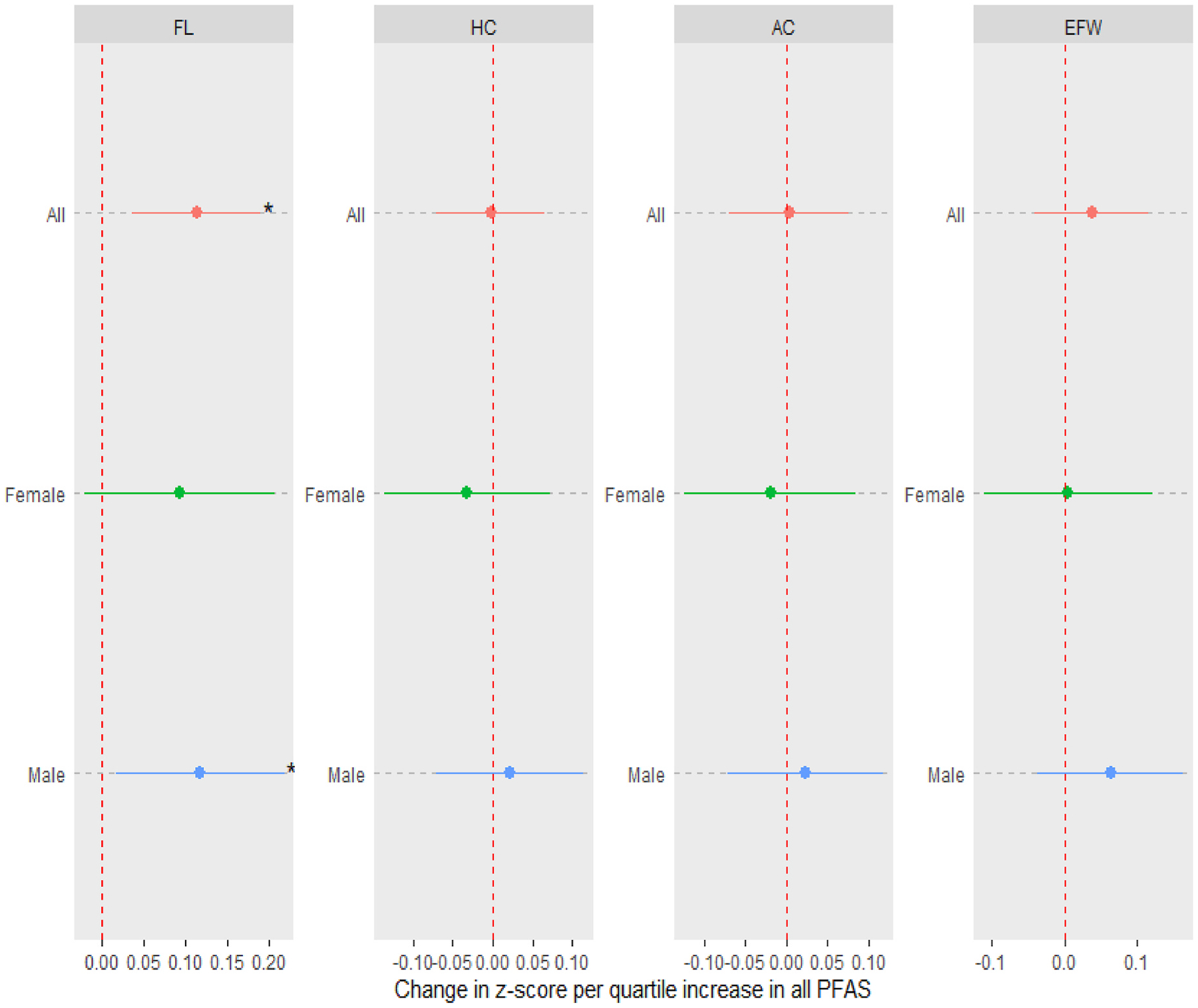
Associations between PFAS mixture (PFOA, PFOS, PFNA, PFHxS, PFDA and PFUA) and standardized ultrasound parameters of fetal growth [femur length (FL), head circumference (HC), abdominal circumference (AC) and estimated fetal weight (EFW)]. Estimates derived from quantile-based g-computation (qgcomp) using 1000 Monte Carlo simulations and 5000 bootstrap samples under a linear model to obtain robust standard errors. Effect estimates (95% confidence intervals) show the change in Z-score (or standard deviation) of each growth measure per quartile increase in PFAS levels. Models for all participants were adjusted for maternal age, prepregnancy BMI, race, education, insurance status, parity, fetal sex, enrollment year, and number of ultrasound scans. Asterisks indicate estimates with confidence intervals excluding the null.

In mixture analyses examining the joint effects of six PFAS compounds on fetal growth, our findings were largely consistent with the single pollutant analysis (Fig. 4, Table S9). For instance, the PFAS mixture was positively associated with FL, with a simultaneous quartile increase in the levels of all PFAS leading to a 0.11 (95% CI: 0.04, 0.19) SD increase in FL Z-score. The joint effect of the PFAS mixture on birthweight Z-score showed positive but non-significant associations in the overall cohort and in females (Table S10).

### 3.5. Sensitivity analyses

Our findings remained robust across multiple sensitivity analyses (Tables S11–S12). For instance, replacing standardized Z-scores with raw fetal growth measurements showed a small but positive increase in femur length (0.23 mm; 95% CI: 0, 0.46) per IQR increase in PFOA, consistent with the positive association observed in our main analysis. Similarly, results remained consistent when excluding participants who reported smoking during pregnancy, when adjusting for GA at PFAS measurement, and when removing extreme ultrasound measurement outliers. Finally, restricting analysis to participants selected as part of the case-control study, i.e., excluding participants from the pilot analyses, or repeating the primary analysis using linear mixed-effects regression models also did not result in any meaningful changes to our primary findings (data not shown).

### 3.6. Exploratory ultrasound fetal growth trajectory modeling

In the LCTM analysis, both the three- and four-class models met minimum statistical criteria outlined in the Methods section (Table S13). We retained the four-class solution because it yielded more interpretable subgroups, including two extreme classes (≈10–15% of the cohort each) (Fig. S6), whereas the three-class solution grouped a larger share of participants into broad medium categories. From the chosen model, class 1 (9.6% of the cohort) showed consistently small growth measurements across all parameters and was labeled “Small”; class 2 (34.9%) demonstrated moderate growth on the lower end of the spectrum and was labeled “Lower-Medium”; class 3 (40.4%) showed “Upper-Medium” growth trajectories, with measurements consistently above the population mean; and class 4 (15.2%) was characterized by initially large measurements with progressively declining HC and FL values over gestation, and was labeled “Large with Declining HC and FL”. The model demonstrated good classification quality, with average posterior probabilities for each class above 0.85 and the relative entropy of 0.77.

Fig. S7 illustrates the distributions of birth and clinical outcomes across the four trajectory classes. As expected, we observed a clear gradient in birthweight Z-score, with the “Small” class having the lowest median scores and the “Large with Declining HC and FL” class having the highest. The “Small” class contained 35.7% of SGA newborns while the “Large with Declining HC and FL” class contained 47.1% of LGA newborns. Within the “Small” class, 38.5% were PTB cases compared to 26.7% in “Lower-Medium”, 28.2% in “Upper-Medium”, and 30.6% in “Large with Declining HC and FL” classes. Additionally, 23.1% and 22.3% of participants in the “Small” class were placental PTB and spontaneous PTB cases, respectively. The “Small” class also had the largest proportion of NICU-admitted newborns (40.8%) compared to other classes. When we examined the associations between each PFAS and class membership, using the combined medium classes as reference, we observed generally positive associations between prenatal PFAS concentrations and membership in both extreme trajectory classes (Fig. S8). Specifically, there was a 35% increased relative risk (RRR: 1.35; 95% CI: 1.00, 1.83) of belonging to the “Large with Declining HC and FL” class per IQR increase in PFOA levels. Similarly, the relative risks of belonging to the “Small” class were also elevated with increasing levels of some PFAS, although these associations included the null.

## 4. Discussion

In this study, we examined associations between prenatal PFAS exposure and measures of fetal growth (via longitudinal ultrasound parameters and birthweight outcomes) among racially and socioeconomically diverse LIFECODES study participants. Consistent with the national trends (CDC, 2024), we observed declining concentrations of all PFAS over time, with six compounds detectable in most participants during the study period. Our findings showed that elevated levels of several PFAS, most consistently PFOA, were linked with modest increases in FL Z-score in the overall cohort, whereas associations with other ultrasound parameters and birthweight Z-score were generally weak or null. Notably, positive associations for several PFAS were more pronounced in participants who identified as African Americans as compared to non-Hispanic Whites or other racial/ethnic groups. We also identified some fetal sex-specific patterns, including a stronger positive association between PFOA and FL Z-score among male fetuses, although these patterns varied by PFAS compounds and growth parameters. Our exploratory latent class trajectory modeling further identified distinct fetal growth profiles with different clinical risk profiles, with higher PFAS levels linked with increased risk of membership in the “Small” and “Large with Declining HC and FL” classes.

The observed positive associations between PFAS and FL Z-scores are consistent with findings from the NICHD Fetal Growth Studies [Ouidir et al. (2020); N = 2284], where early-pregnancy (median GA = 12 weeks) PFOA, PFNA, PFHxS, and PFHP levels were modestly linked to longer FL across gestation. In contrast, a few cohorts with smaller sample sizes have reported null or inverse patterns. In the predominantly Hispanic MADRES cohort [Peterson et al. (2022); N = 335], mid-gestation (~21 weeks) PFOA was associated with smaller third-trimester HC among women with higher perceived stress, while no associations were seen in the lower-stress group, and the other measures of fetal growth were not analyzed. In Spain, Costa et al. (2019) (INMA Project) reported inverse associations of several PFAS (GA ~ 13.5 weeks) with FL, AC, and EFW, especially among smokers, although no associations were reported among non-smokers. Similarly, another Spanish study in the BiSC cohort [Knox et al. (2024); N = 747] found slight reductions in EFW between 32 and 37 weeks of gestation in relation to higher PFAS levels in late pregnancy (~32 weeks), along with a suggestive positive association between PFOSA and FL. Finally, in a recent study based in the Changsha Hospital in Hunan Province, China (N = 352), Yang and colleagues (Yang et al., 2025) reported inverse associations between concentrations of several legacy PFAS (GA ~ 11–13 weeks) and ultrasound measures, while the newer PFAS were positively associated, including with FL. The reasons for discrepancies across these studies are likely multifactorial, some of which include differences in study population characteristics (race/ethnicity, socioeconomic status, genetics, baseline health, co-exposures like stress or smoking), timing of exposure or ultrasound assessment, levels and mixtures of PFAS exposure (varying geographically and temporally), outcome assessment methods (e.g., internal vs. external growth standards), and analytical approaches (e.g., covariate adjustment, exposure modeling like continuous vs. dichotomized). Interestingly, while Peterson et al. (Peterson et al., 2022) reported a statistically significant inverse association between PFOA and HC Z-scores among predominantly Hispanic participants, our own analysis within the Hispanic subgroup found an inverse but non-significant association for PFOA, and largely null associations between other PFAS and ultrasound measures. We did, however, note suggestive inverse trends between some PFAS and birthweight Z-score in this subgroup. These inconsistencies, even within populations with similar (but broad) ethnic categorization, underscore the challenge of comparing epidemiological findings and the importance of considering cohort-specific factors that influence the associations.

The biological mechanism underlying the modest elongation of the femur in relation to increased levels of PFAS is not fully clear; nonetheless, currently existing evidence suggests that these chemicals may affect biological pathways crucial for fetal growth and development. Within the maternal system, PFAS have been shown to disrupt endocrine functions, particularly thyroid hormone homeostasis, with several studies reporting PFAS-related shifts in maternal T3, T4, or TSH levels during pregnancy (Coperchini et al., 2024; Ballesteros et al., 2017). Since the fetus is fully dependent on maternal thyroid hormones for skeletal development in early gestation (Fisher, 1997), such alterations could adversely affect hormone delivery and normal skeletal growth (Shields et al., 2011; Burrow et al., 1994). Additionally, PFAS have been shown to interact with peroxisome proliferator-activated receptors (PPARs), which regulate fatty acid and glucose metabolism as well as insulin sensitivity (Szilagyi et al., 2020), and may affect placental nutrient transfer to the fetus. Beyond the maternal effects, these compounds also readily cross the placental barrier (Appel et al., 2022), and may directly influence fetal bone growth. For instance, a few experimental and epidemiological studies have indicated that PFAS can interfere with bone cell differentiation and function by altering osteoblast and osteoclast activity, and potentially impair bone mineralization by competing with vitamin D for its receptor binding in osteoblasts (Turan, 2021; Koskela et al., 2016; Wang et al., 2024). Although most of these pathways point toward disruption of normal bone development, generally suggesting attenuation rather than enhancement of growth; our results, considered alongside the exploratory LCTM analysis, indicate potential tissue-specific and time-dependent effects. For instance, PFAS were modestly associated with greater FL on average across gestation, together with a trajectory characterized by later restriction of HC and FL.

Our findings for birthweight outcomes were mixed. The null results for birthweight Z-score were not unexpected, as we observed similar patterns in a previous analysis of this cohort with a subset of participants (Siwakoti et al., 2023a). Interestingly, we found decreased odds of SGA in relation to several PFAS, which diverges from some existing studies that have associated higher prenatal PFAS exposure with reduced birthweight or increased odds of SGA (Gao et al., 2021; Bach et al., 2015; Wright et al., 2023; Gui et al., 2022; Fu et al., 2025). A clear biological rationale for this finding is difficult to establish, and as with ultrasound measures, methodological factors, including the potential for chance findings and residual confounding by unmeasured dietary factors, may have contributed to these associations. Furthermore, the associations between certain PFAS and LGA appeared to be potentially fetal sex-specific, with suggestive evidence of increased odds of LGA among females. This sex-specific observation needs cautious interpretation given the relatively small number of LGA cases in our study, but it highlights the need for further investigation into underlying metabolic (Braun et al., 2022; Svoboda et al., 2022; Everson et al., 2025; Alur, 2019), epigenetic (Svoboda et al., 2022; Petroff et al., 2023), endocrine (Braun et al., 2022), or placental (Rosenfeld, 2015) mechanisms that might differentially impact male and female fetal growth. The few studies that have examined PFAS and LGA in a sex-specific manner also report inconsistent findings (Gao et al., 2021; Padula et al., 2023; Whitworth et al., 2012). For instance, while Padula et al. (2023) reported lower odds of LGA for some PFAS in the overall cohort, the inverse effect was stronger in females as compared to males. These discrepancies in birthweight effect estimates across cohorts likely reflect heterogeneity in study design, exposure timing, population characteristics, and analytical approaches.

For fetal growth measures both in utero and at birth, we observed a tendency towards generally more positive associations with PFAS among African American participants compared to other racial/ethnic groups. This trend is partially in line with Ouidir et al. (2020), who similarly reported more positive associations among Black women, although it contrasts with results from Eick et al. (2024), who documented null or non-significant inverse associations between PFAS and birthweight Z-score in the African American birth cohort in Atlanta. The race/ethnicity-specific differences we observed may stem from a variety of factors, including possible differences in genetics, dietary patterns, or baseline metabolic health that might modify individual responses to environmental exposures. Additionally, while our models adjusted for several socioeconomic indicators, race in our study context may serve as a proxy for unmeasured personal experiences (Lett et al., 2022), potentially including differential exposure to chronic stressors or environmental co-contaminants, which might interact with PFAS independently and affect biological pathways related to fetal growth through mechanisms that differ between groups.

Our exploratory latent class analysis identified distinct patterns of fetal growth that capture longitudinal heterogeneity not detectable through single time-point measures. Unlike previous applications of LCTM focused on other environmental exposures in relation to extreme birth outcomes like SGA (Bommarito et al., 2023b) or LGA (Bommarito et al., 2023a), we applied LCTM across all participants, resulting in four trajectory classes with varying clinical risk profiles. This suggests that LCTM, even applied to the entire cohort, may be useful for investigating how environmental exposures relate to diverse developmental trajectories, and generate hypotheses relevant for fetal growth or other longitudinal health outcomes that would not be evident if only the traditional summary measures of fetal growth such as birthweight (or SGA/LGA) were used.

Our study has several strengths. First, we used a relatively large and diverse cohort from the LIFECODES study, which enhanced the generalizability of our findings, and allowed us to explore effect modification by fetal sex and maternal race. Second, the extended recruitment period spanning more than a decade (2006–2019) allowed us to assess temporal trends in PFAS exposure within LIFECODES and provided substantial exposure contrast for our analysis. Third, the use of repeated ultrasound measurements throughout pregnancy allowed for a dynamic assessment of fetal growth trajectories, offering advantages over studies that rely on single time-point measurements. Fourth, we measured multiple PFAS prospectively in early pregnancy, which allowed us to maintain clear temporality between exposures and subsequent fetal growth measures. The earlier sample collection also reduced the likelihood of confounding due to increased plasma volume expansion and changes in glomerular filtration rate that can affect PFAS levels (Sagiv et al., 2017). Fifth, our use of internal reference standards used to calculate Z-scores, derived from the same obstetric population at BWH (Cantonwine et al., 2016), likely reduced potential outcome misclassification and strengthened the validity of our growth assessments. Finally, we used rigorous statistical methods and incorporated multiple sensitivity analyses, which ensured a comprehensive evaluation and robustness of our findings.

Our study also has some limitations that should be considered when interpreting its findings. First, given the observational nature of the study, our findings are not necessarily causal. While we adjusted for several potential confounders, residual confounding from unmeasured factors, including maternal nutritional status, genetic factors, or co-exposures to other environmental chemicals cannot be ruled out. We did not have comprehensive data on maternal dietary supplement intake (such as vitamin D, calcium, folate, multivitamin, and iron) across all participants, which could be relevant given that more than 75% of pregnant women use one or more supplements that might influence fetal growth (Jun et al., 2020). However, in sensitivity analyses conducted on a subset of participants with available information, adjusting for supplement use did not meaningfully influence our findings (data not shown). In addition, we did not have data on seafood intake, which could contribute to residual confounding, particularly for directionally positive associations, because seafood can be both a source of PFAS exposure and a source of nutrients that may independently support fetal growth. Second, we measured only legacy PFAS compounds with available laboratory standards at the time of analysis, potentially missing effects of newer PFAS or their replacements. However, the PFAS compounds we measured remain highly prevalent and likely represent a major component of PFAS exposure currently experienced by the general U.S. population (CDC, 2024). Third, while we standardized ultrasound measures according to established clinical protocols, they inherently contain some degree of measurement error (Dudley, 2005), particularly for calculated outcomes like EFW, which could add variability or potentially attenuate observed associations. Fourth, because we tested multiple hypotheses, some observed associations may reflect chance findings and should be interpreted cautiously, in the context of the broader pattern of evidence for the relevant exposure and outcome. Finally, while we found statistically significant associations between some PFAS and fetal growth measures, the clinical significance of the modest effect sizes (e.g., a 0.10 SD increase for PFOA and FL) that we observed remains unclear. While a deviation of this magnitude may fall within normal variation, even a small population-level shift may reflect meaningful biological perturbations, particularly in susceptible subgroups, and warrants further investigation.

## 5. Conclusions

In the racially diverse LIFECODES cohort, we examined associations between prenatal PFAS exposure and measures of fetal growth both in utero and at birth. We observed a modest increase in FL Z-score in relation to some PFAS, with associations varying by fetal sex and maternal race in certain instances. Furthermore, we found some evidence of increased odds of LGA, specifically among females, and decreased odds of SGA related to select PFAS, though these associations were not consistent across all PFAS compounds or the examined subgroups. Overall, our results suggest that elevated prenatal PFAS exposure may be associated with subtle alterations in fetal growth, and future research should examine underlying biological mechanisms, clinical implications, and the influence of modifying factors such as maternal diet and psychosocial stress on the observed associations.

## Supplementary Material

1

## Figures and Tables

**Table 1:** Demographic, behavioral, and clinical characteristics of study participants.

	Overall (N = 1220)
Maternal age (y)	
Median (Q1, Q3)	32.8 (28.8, 36.2)
Maternal Prepregnancy BMI (kg/m^2^)	
Median (Q1, Q3)	25.0 (21.9, 29.5)
Missing	19 (1.6%)
Maternal race	
African American	179 (14.7%)
Asian	84 (6.9%)
Non-Hispanic White	693 (56.8%)
Hispanic	198 (16.2%)
Other	66 (5.4%)
Maternal educational attainment	
High school or less	160 (13.1%)
Some college or technical degree	218 (17.9%)
Bachelor’s degree or more	817 (67.0%)
Missing	25 (2.0%)
Insurance status	
Private insurance/HMO	894 (73.3%)
Self-pay or Medicaid/Mass Health	310 (25.4%)
Missing	16 (1.3%)
Nulliparous	
No	696 (57.0%)
Yes	524 (43.0%)
Smoking during pregnancy	
No	1136 (93.1%)
Yes	83 (6.8%)
Missing	1 (0.1%)
Alcohol during pregnancy	
No	1123 (92.0%)
Yes	82 (6.7%)
Missing	15 (1.2%)
Fetal sex	
Female	567 (46.5%)
Male	649 (53.2%)
Missing	4 (0.3%)
Preterm birth	
No	865 (70.9%)
Yes	355 (29.1%)
Categorized birthweight status	
SGA	116 (9.5%)
AGA	982 (80.5%)
LGA	114 (9.3%)
Missing	8 (0.7%)
Year of sample collection^1^	
2006	47 (3.9%)
2007	264 (21.6%)
2008	203 (16.6%)
2009	34 (2.8%)
2010	59 (4.8%)
2011	109 (8.9%)
2012	110 (9.0%)
2013	102 (8.4%)
2014	45 (3.7%)
2015	69 (5.7%)
2016	76 (6.2%)
2017+	102 (8.4%)
Number of ultrasound scans per participant^1^	
1	134 (11.0%)
2	169 (13.9%)
3	176 (14.4%)
4	216 (17.7%)
5	193 (15.8%)
6	143 (11.7%)
7	91 (7.5%)
8	49 (4.0%)
9	28 (2.3%)
10+	21 (1.8%)

**Note:** All estimates are unweighted.

1 Participants enrolled from 2017 to 2019 were grouped in a single category due to relatively smaller number of participants in each of those years. Similarly, participants with 10 or more scans were also grouped. Abbreviations: SGA, small-for-gestational age; AGA, average-for-gestational age; LGA, large-for-gestational age.

**Table 2:** Distribution of plasma PFAS concentrations among study participants during the study period (2006–2019) (N = 1220).

PFAS	Detection rate (%)	Median (Q1, Q3) (ng/mL)
PFOA	94.10	1.69 (1.00, 2.64)
PFOS	100.00	4.66 (2.86, 7.45)
PFNA	99.26	0.67 (0.44, 0.95)
PFHxS	98.03	0.71 (0.39, 1.22)
PFDA	84.75	0.21 (0.13, 0.31)
PFUA	68.20	0.16 (0.07, 0.28)

Note: MPAH, PFHP and PFOSA were detected in 45.33%, 18.85% and 0.33% of samples, respectively, and were excluded from further analyses.

## Data Availability

The authors do not have permission to share data.

## Appendix A. Supplementary data

Supplementary data to this article can be found online at https://doi.org/10.1016/j.envres.2026.124279.
