# Supplementary material for: Associations between prenatal per- and polyfluoroalkyl substances (PFAS) exposure and fetal growth measurements in utero and at birth in the LIFECODES cohort: 2006–2019
Source: Environ Res. Author manuscript; Available in PMC 2026 Jun 3. (PMC13233116; doi:10.1016/j.envres.2026.124279)
Supplement: 1 [file NIHMS2180156-supplement-1.docx]

**List of Figures**

[Figure S1. Directed acyclic graph (DAG) used to select adjustment sets for modeling. Covariates represented by white spheres were included as primary adjustment variables. All models were adjusted for the year of enrollment to account for secular trends in PFAS levels over time. Fetal sex was included as a precision variable to improve model efficiency. Gestational age at ultrasound measurement was not included as a covariate in models using Z-scored ultrasound outcomes, as these scores inherently account for gestational age. Models for ultrasound parameters were also adjusted for the number of scans, as a higher number of scans may indicate pregnancies with clinical complications that could potentially influence fetal growth. 3](#_Toc223211420)

[Figure S2. Changes in PFAS concentrations over time. The distributions are not weighted using case-control weights. 4](#_Toc223211421)

[Figure S3. Spearman correlation coefficients between different PFAS compounds. 4](#_Toc223211422)

[Figure S4. Fetal growth trajectories based on raw measurements of FL, HC, AC, and EFW. FL, HC, and AC were measured in mm, while EFW was estimated in gram. 5](#_Toc223211423)

[Figure S5. Spearman correlation coefficients between Z-scored fetal growth measurements. 5](#_Toc223211424)

[Figure S6. Latent class trajectories of standardized fetal growth measurements based on multivariate trajectory analysis. A four-class solution was selected based on fit criteria, including AIC, BIC, posterior probability, and entropy. All groups comprised at least 5% of participants (N = 1,220). The Y-axis represents Z-scored measurements, and the X-axis represents gestational age in weeks. The four trajectory groups were labeled as Small, Lower-Medium, Upper-Medium, and Large with Declining HC and FL. The average posterior probability for group 1, 2, 3, 4 were 0.91, 0.86, 0.86, and 0.88, respectively. The entropy of the chosen model was 0.77 (see Table S13 for details). 6](#_Toc223211425)

[Figure S7. Distributions of observed birth/clinical outcomes across different latent classes. Abbreviations: PTB: preterm birth; Spont.: spontaneous; Plac.: placental; BW: birthweight; NICU: neonatal intensive care unit. 7](#_Toc223211426)

[Figure S8. Associations between prenatal PFAS exposure and latent class trajectories were estimated using multinomial logistic regression models. Relative risk ratios (RRRs) and 95% CIs represent the relative risk of being assigned to each class compared to the Medium class. For the purpose of statistical modeling, Lower-Medium and Upper-Medium classes were combined into a single Medium class, and used as a reference category. All models were adjusted for maternal age, prepregnancy BMI, race, education, insurance status, parity, enrollment year, and fetal sex. Analysis was based on the complete-case data. The sample size for each class was 127 (Small), 874 (Medium), and 172 (Large with Declining HC and FL). 8](#_Toc223211427)

**List of Tables**

[Table S1. Distributions of PFAS concentrations [median (Q1, Q3)] by demographic, behavioral, and clinical characteristics of study participants (N = 1220). 9](#_Toc223211430)

[Table S2. Distributions of fetal growth measurements [median (Q1, Q3)] by demographic, behavioral, and clinical characteristics of study participants (N = 1220). 11](#_Toc223211431)

[Table S3. Distributions of mean gestational age (GA) by scan number. 13](#_Toc223211432)

[Table S4. Associations between prenatal PFAS exposure and ultrasound measures of fetal growth. 14](#_Toc223211433)

[Table S5. P-values for the interaction term related to fetal sex. 15](#_Toc223211434)

[Table S6. Associations between prenatal PFAS exposure and ultrasound measures of fetal growth stratified by maternal race. 16](#_Toc223211435)

[Table S7. Associations between prenatal PFAS exposure and birthweight Z-scores among all participants and across different subsets. 17](#_Toc223211436)

[Table S8. Associations between prenatal PFAS exposure and categorized measures of extreme birthweight Z-scores. 17](#_Toc223211437)

[Table S9. Associations between prenatal PFAS mixture and ultrasound measures of fetal growth among all participants and across different subsets. 18](#_Toc223211438)

[Table S10. Associations between prenatal PFAS mixture and birthweight Z-scores among all participants and across different subsets. 18](#_Toc223211439)

[Table S11. Sensitivity analyses related to the associations between prenatal PFAS exposure and ultrasound measures of fetal growth. 20](#_Toc223211440)

[Table S12. Associations between prenatal PFAS exposure and birthweight (or Z-scores) among all participants and across different subsets. 20](#_Toc223211441)

[Table S13. Model fit statistics for latent class trajectory models (N = 1220). 21](#_Toc223211442)


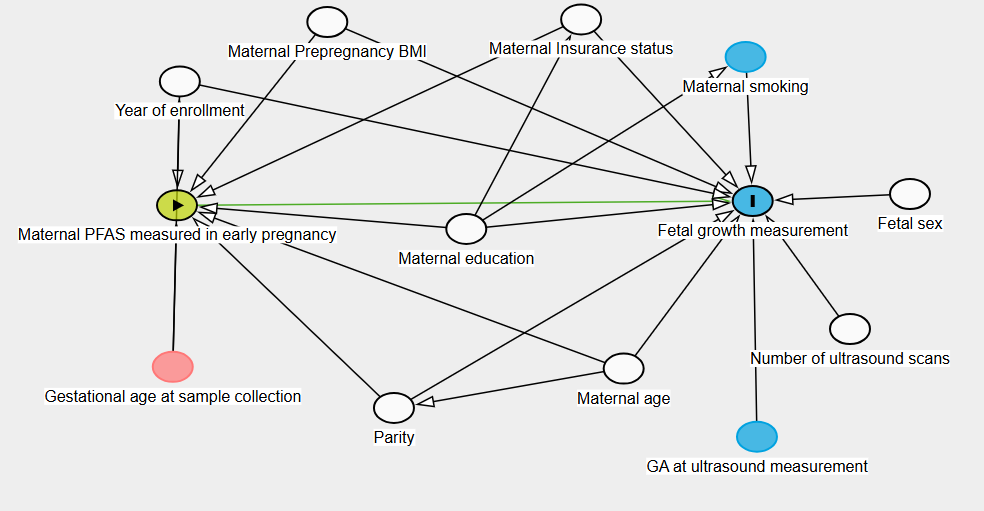


Figure S1. Directed acyclic graph (DAG) used to select adjustment sets for modeling. Covariates represented by white spheres were included as primary adjustment variables. All models were adjusted for the year of enrollment to account for secular trends in PFAS levels over time. Fetal sex was included as a precision variable to improve model efficiency. Gestational age at ultrasound measurement was not included as a covariate in models using Z-scored ultrasound outcomes, as these scores inherently account for gestational age. Models for ultrasound parameters were also adjusted for the number of scans, as a higher number of scans may indicate pregnancies with clinical complications that could potentially influence fetal growth.


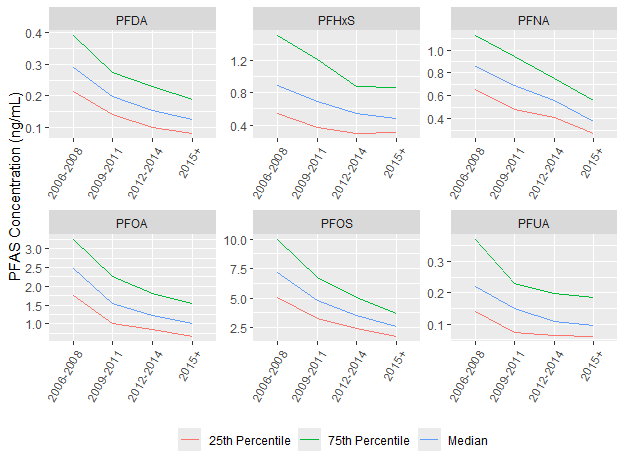


Figure S2. Changes in PFAS concentrations over time. The distributions are not weighted using case-control weights.


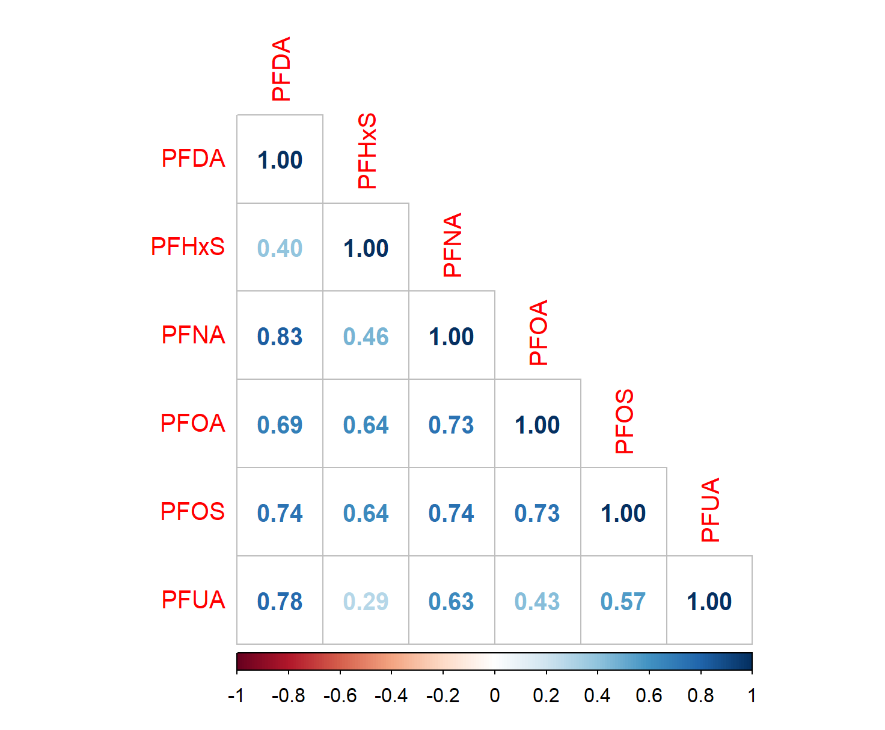


Figure S3. Spearman correlation coefficients between different PFAS compounds.


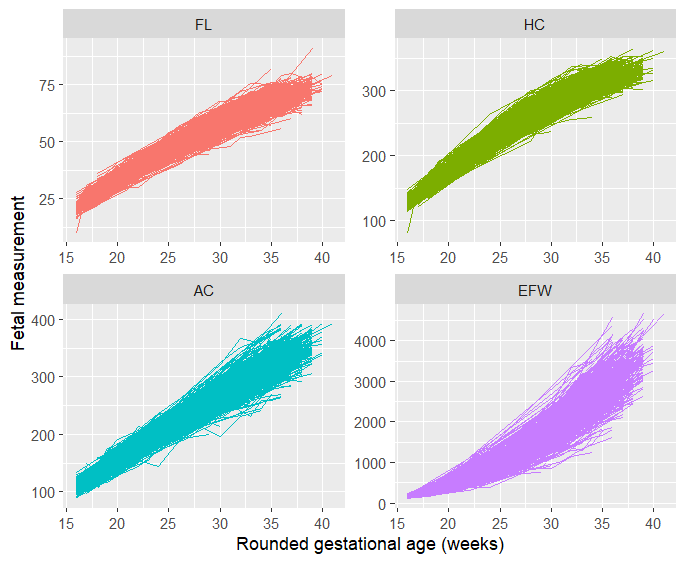


Figure S4. Fetal growth trajectories based on raw measurements of FL, HC, AC, and EFW. FL, HC, and AC were measured in mm, while EFW was estimated in grams.


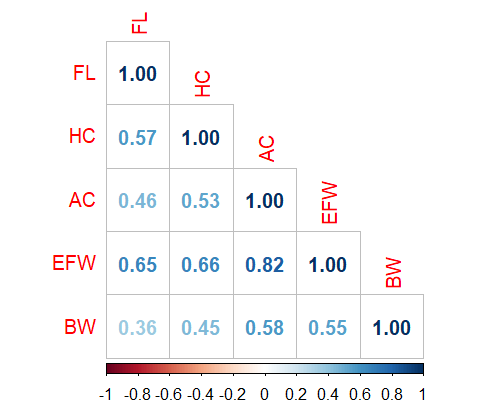


Figure S5. Spearman correlation coefficients between Z-scored fetal growth measurements.

FL


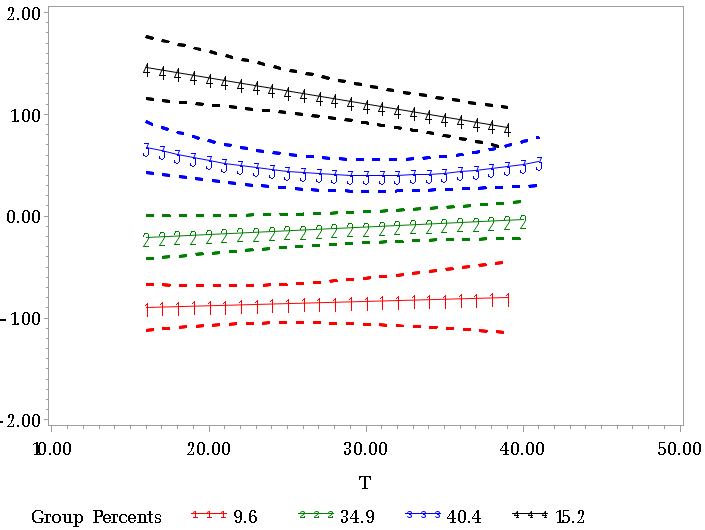


HC


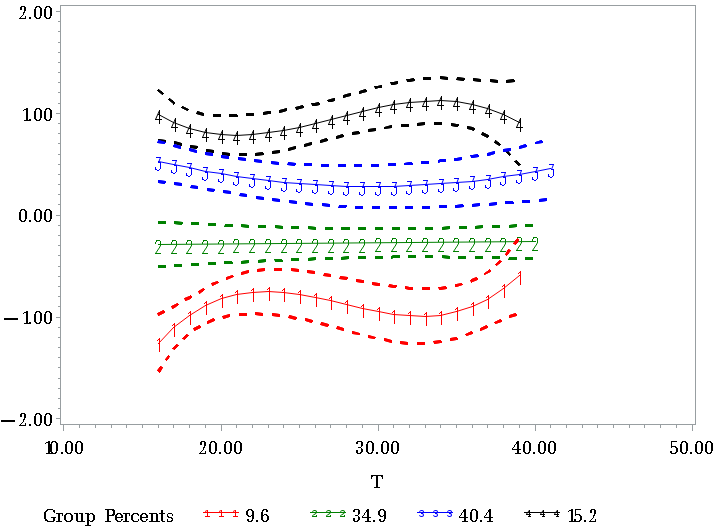


AC

FL


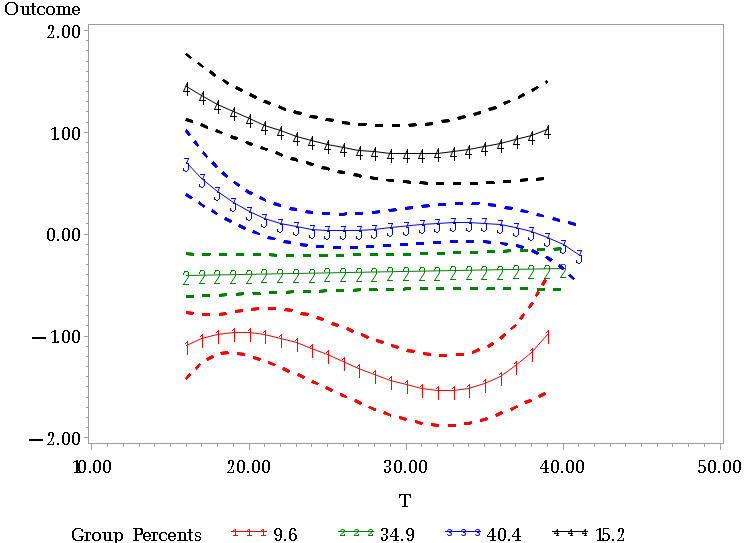


Figure S6. Latent class trajectories of standardized fetal growth measurements based on multivariate trajectory analysis. A four-class solution was selected based on fit criteria, including AIC, BIC, posterior probability, and entropy. All groups comprised at least 5% of participants (N = 1,220). The Y-axis represents Z-scored measurements, and the X-axis represents gestational age in weeks. The four trajectory groups were labeled as Small, Lower-Medium, Upper-Medium, and Large with Declining HC and FL. The average posterior probabilities for group 1, 2, 3, 4 were 0.91, 0.86, 0.86, and 0.88, respectively. The entropy of the chosen model was 0.77 (see Table S13 for details).


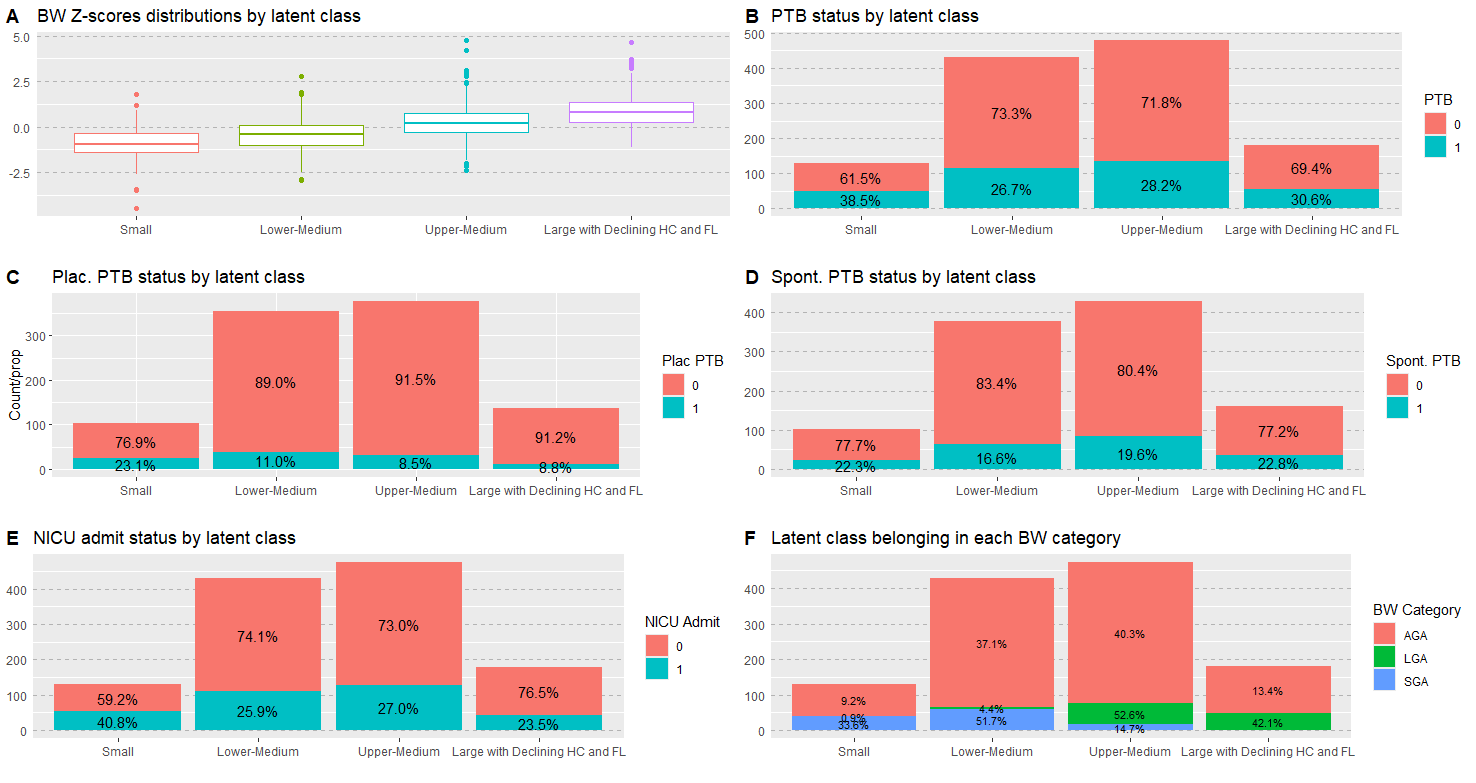


Figure S7. Distributions of observed birth/clinical outcomes across different latent classes. Abbreviations: PTB: preterm birth; Spont.: spontaneous; Plac.: placental; BW: birthweight; NICU: neonatal intensive care unit.


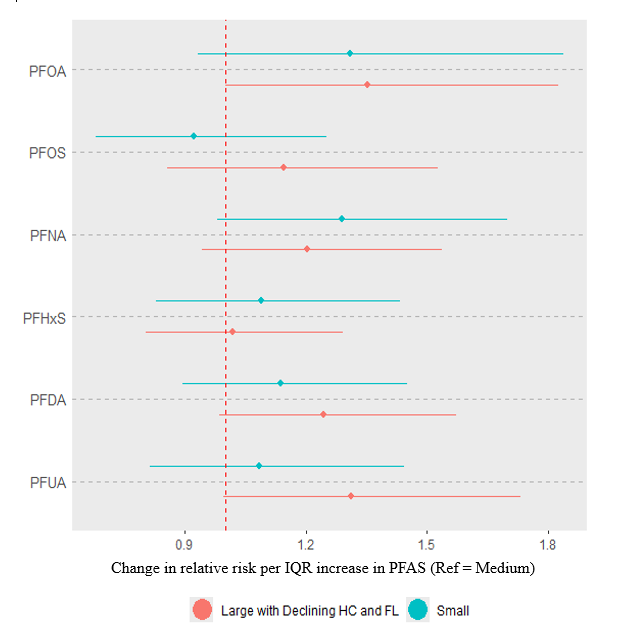


Figure S8. Associations between prenatal PFAS exposure and latent class trajectories were estimated using multinomial logistic regression models. Relative risk ratios (RRRs) and 95% CIs represent the relative risk of being assigned to each class compared to the Medium class. For the purpose of statistical modeling, Lower-Medium and Upper-Medium classes were combined into a single Medium class and used as a reference category. All models were adjusted for maternal age, prepregnancy BMI, race, education, insurance status, parity, enrollment year, and fetal sex. Analysis was based on the complete-case data. The sample size for each class was 127 (Small), 874 (Medium), and 172 (Large with Declining HC and FL).

Table S1. Distributions of PFAS concentrations [median (Q1, Q3)] by demographic, behavioral, and clinical characteristics of study participants (N = 1220).

|  | **N** | **PFOA** | **PFOS** | **PFNA** | **PFHxS** | **PFDA** | **PFUA** |
| --- | --- | --- | --- | --- | --- | --- | --- |
| **Maternal age (y)** |  |  |  |  |  |  |  |
| Above Median (> 32.8) | 608 | 1.65 (1, 2.58) | 4.66 (2.96, 7.46) | 0.65 (0.44, 0.91) | 0.72 (0.38, 1.22) | 0.21 (0.13, 0.32) | 0.17 (0.09, 0.31) |
| Below Median (≤ 32.8) | 612 | 1.73 (1.01, 2.73) | 4.69 (2.83, 7.39) | 0.69 (0.44, 0.99) | 0.7 (0.4, 1.21) | 0.21 (0.13, 0.3) | 0.15 (0.07, 0.25) |
| **Maternal Prepregnancy BMI** |  |  |  |  |  |  |  |
| BMI ≤ 25 | 605 | 1.71 (1.06, 2.7) | 4.89 (3.02, 7.7) | 0.67 (0.46, 0.98) | 0.72 (0.41, 1.29) | 0.22 (0.14, 0.32) | 0.19 (0.11, 0.32) |
| 25 < BMI ≤ 30 | 313 | 1.77 (1, 2.72) | 5.01 (3.04, 7.62) | 0.69 (0.45, 0.98) | 0.72 (0.37, 1.16) | 0.22 (0.14, 0.32) | 0.16 (0.07, 0.28) |
| BMI > 30 | 283 | 1.54 (0.84, 2.36) | 4.02 (2.41, 6.71) | 0.62 (0.39, 0.89) | 0.65 (0.37, 1.15) | 0.18 (0.11, 0.28) | 0.11 (0.07, 0.18) |
| Missing | 19 | 1.59 (0.96, 2.67) | 3.94 (2.52, 7.04) | 0.71 (0.45, 0.77) | 0.55 (0.24, 1.07) | 0.17 (0.12, 0.26) | 0.11 (0.07, 0.2) |
| **Maternal race/ethnicity** |  |  |  |  |  |  |  |
| African American | 179 | 1.44 (0.87, 2.26) | 4.65 (2.79, 7.53) | 0.7 (0.46, 0.96) | 0.57 (0.33, 0.96) | 0.24 (0.14, 0.33) | 0.16 (0.1, 0.27) |
| Asian | 84 | 1.9 (1.04, 2.75) | 6.61 (3.23, 9.83) | 0.86 (0.46, 1.21) | 0.72 (0.37, 1.27) | 0.27 (0.15, 0.47) | 0.28 (0.13, 0.54) |
| Non-Hispanic White | 693 | 1.84 (1.09, 2.79) | 4.98 (3.04, 7.7) | 0.65 (0.45, 0.91) | 0.83 (0.48, 1.38) | 0.21 (0.14, 0.3) | 0.16 (0.08, 0.28) |
| Hispanic | 198 | 1.47 (0.82, 2.37) | 3.62 (2.19, 5.53) | 0.64 (0.38, 1) | 0.45 (0.26, 0.74) | 0.18 (0.1, 0.28) | 0.12 (0.07, 0.2) |
| Other | 66 | 1.57 (0.96, 2.26) | 4.86 (2.83, 7.1) | 0.67 (0.37, 0.89) | 0.78 (0.36, 1.03) | 0.21 (0.13, 0.3) | 0.16 (0.08, 0.26) |
| **Maternal educational attainment** |  |  |  |  |  |  |  |
| High school or less | 160 | 1.57 (0.86, 2.39) | 4.35 (2.72, 6.78) | 0.67 (0.43, 0.99) | 0.56 (0.36, 0.93) | 0.19 (0.12, 0.3) | 0.13 (0.07, 0.19) |
| Some college or technical degree | 218 | 1.58 (0.9, 2.4) | 4.32 (2.45, 6.83) | 0.69 (0.38, 0.99) | 0.57 (0.32, 1.05) | 0.2 (0.11, 0.3) | 0.14 (0.07, 0.23) |
| Bachelor's degree or more | 817 | 1.75 (1.07, 2.75) | 4.9 (3.02, 7.84) | 0.65 (0.46, 0.95) | 0.77 (0.42, 1.34) | 0.22 (0.14, 0.31) | 0.17 (0.09, 0.31) |
| Missing | 25 | 1.44 (0.92, 2.14) | 4.14 (2.33, 6.22) | 0.72 (0.34, 0.84) | 0.6 (0.38, 1.05) | 0.25 (0.12, 0.29) | 0.19 (0.1, 0.25) |
| **Health insurance** |  |  |  |  |  |  |  |
| Private insurance/HMO | 894 | 1.81 (1.08, 2.75) | 5 (3.03, 8) | 0.67 (0.47, 0.96) | 0.78 (0.44, 1.31) | 0.22 (0.14, 0.32) | 0.17 (0.09, 0.31) |
| Self-pay or Medicaid/Mass Health | 310 | 1.34 (0.82, 2.13) | 4.05 (2.31, 5.86) | 0.64 (0.38, 0.91) | 0.5 (0.28, 0.86) | 0.19 (0.11, 0.27) | 0.13 (0.07, 0.2) |
| Missing | 16 | 2.85 (1.95, 3.35) | 6.02 (3.79, 9.21) | 0.97 (0.74, 1.18) | 0.96 (0.58, 1.31) | 0.3 (0.25, 0.4) | 0.19 (0.15, 0.21) |
| **Parity** |  |  |  |  |  |  |  |
| Parous | 696 | 1.37 (0.87, 2.23) | 4.18 (2.74, 6.71) | 0.61 (0.41, 0.89) | 0.58 (0.35, 0.97) | 0.2 (0.12, 0.29) | 0.15 (0.07, 0.25) |
| Nulliparous | 524 | 2.11 (1.31, 3.03) | 5.49 (3.23, 8.6) | 0.74 (0.5, 1.01) | 0.86 (0.48, 1.41) | 0.24 (0.14, 0.33) | 0.17 (0.09, 0.32) |
| **Smoking during pregnancy** |  |  |  |  |  |  |  |
| No | 1136 | 1.68 (1, 2.64) | 4.66 (2.95, 7.54) | 0.67 (0.44, 0.95) | 0.71 (0.39, 1.23) | 0.21 (0.13, 0.31) | 0.16 (0.08, 0.29) |
| Yes | 83 | 1.72 (0.94, 2.63) | 4.78 (2.75, 6.7) | 0.67 (0.42, 0.98) | 0.67 (0.37, 1.13) | 0.18 (0.11, 0.26) | 0.12 (0.07, 0.2) |
| Missing | 1 | 2.93 (2.93, 2.93) | 3.34 (3.34, 3.34) | 0.74 (0.74, 0.74) | 0.71 (0.71, 0.71) | 0.11 (0.11, 0.11) | 0.1 (0.1, 0.1) |
| **Alcohol during pregnancy** |  |  |  |  |  |  |  |
| No | 1123 | 1.71 (1, 2.65) | 4.66 (2.84, 7.49) | 0.67 (0.44, 0.95) | 0.72 (0.39, 1.22) | 0.21 (0.13, 0.31) | 0.16 (0.07, 0.28) |
| Yes | 82 | 1.51 (1.05, 2.25) | 4.67 (3.3, 6.77) | 0.65 (0.49, 0.93) | 0.7 (0.45, 1.23) | 0.21 (0.13, 0.29) | 0.17 (0.08, 0.29) |
| Missing | 15 | 1.59 (1.13, 2.83) | 3.94 (3.25, 7.48) | 0.68 (0.47, 0.86) | 0.65 (0.4, 1.07) | 0.25 (0.14, 0.31) | 0.19 (0.1, 0.22) |
| **Fetal sex** |  |  |  |  |  |  |  |
| Female | 567 | 1.63 (0.94, 2.47) | 4.65 (2.84, 7.29) | 0.65 (0.45, 0.93) | 0.68 (0.37, 1.17) | 0.21 (0.13, 0.3) | 0.16 (0.07, 0.27) |
| Male | 649 | 1.74 (1.06, 2.74) | 4.77 (2.97, 7.7) | 0.67 (0.44, 0.97) | 0.72 (0.4, 1.23) | 0.22 (0.13, 0.32) | 0.16 (0.08, 0.29) |
| Missing | 4 | 2.37 (1.87, 3.17) | 5.38 (3.69, 6.37) | 0.8 (0.61, 0.99) | 1.18 (0.56, 2.04) | 0.26 (0.16, 0.43) | 0.12 (0.07, 0.22) |
| **Preterm birth** |  |  |  |  |  |  |  |
| No | 865 | 1.75 (1.04, 2.7) | 4.8 (2.94, 7.54) | 0.67 (0.46, 0.96) | 0.72 (0.4, 1.25) | 0.22 (0.14, 0.31) | 0.16 (0.08, 0.28) |
| Yes | 355 | 1.55 (0.93, 2.42) | 4.39 (2.78, 6.97) | 0.66 (0.42, 0.93) | 0.65 (0.37, 1.13) | 0.2 (0.13, 0.29) | 0.15 (0.07, 0.27) |
| **Categorized birthweight status** |  |  |  |  |  |  |  |
| SGA | 116 | 1.77 (1.07, 2.72) | 4.87 (2.66, 7.72) | 0.68 (0.49, 1.03) | 0.64 (0.34, 1.29) | 0.22 (0.14, 0.34) | 0.16 (0.1, 0.28) |
| AGA | 982 | 1.65 (0.99, 2.59) | 4.64 (2.85, 7.45) | 0.67 (0.44, 0.95) | 0.71 (0.39, 1.22) | 0.21 (0.13, 0.3) | 0.16 (0.07, 0.28) |
| LGA | 114 | 1.85 (1.09, 2.8) | 4.75 (3.15, 7.24) | 0.64 (0.41, 1.01) | 0.78 (0.4, 1.13) | 0.22 (0.14, 0.35) | 0.17 (0.07, 0.27) |
| Missing | 8 | 1.18 (1.04, 2.32) | 4.75 (3.85, 6.37) | 0.68 (0.54, 0.85) | 0.7 (0.5, 0.98) | 0.19 (0.17, 0.25) | 0.18 (0.12, 0.25) |
| **Year of sample collection** |  |  |  |  |  |  |  |
| 2006 | 47 | 2.53 (1.84, 3.16) | 8.45 (5.45, 10.32) | 0.8 (0.61, 1.33) | 0.91 (0.51, 1.36) | 0.3 (0.21, 0.37) | 0.21 (0.13, 0.29) |
| 2007 | 264 | 2.7 (1.8, 3.42) | 7.99 (5.51, 11.29) | 0.9 (0.69, 1.2) | 0.98 (0.6, 1.75) | 0.3 (0.22, 0.41) | 0.26 (0.15, 0.4) |
| 2008 | 203 | 2.26 (1.68, 3) | 6.36 (4.63, 8.46) | 0.81 (0.6, 1.05) | 0.83 (0.48, 1.3) | 0.27 (0.21, 0.36) | 0.2 (0.14, 0.3) |
| 2009 | 34 | 1.73 (1.29, 2.59) | 5.73 (3.93, 7.68) | 0.75 (0.59, 0.96) | 0.8 (0.46, 1.33) | 0.21 (0.15, 0.3) | 0.14 (0.1, 0.22) |
| 2010 | 59 | 1.62 (1.09, 2.22) | 5.14 (3.44, 6.92) | 0.72 (0.58, 1) | 0.73 (0.43, 1.34) | 0.23 (0.16, 0.28) | 0.16 (0.08, 0.25) |
| 2011 | 109 | 1.36 (0.92, 2.19) | 4.11 (2.94, 5.77) | 0.61 (0.43, 0.91) | 0.68 (0.35, 1.1) | 0.19 (0.14, 0.24) | 0.15 (0.07, 0.22) |
| 2012 | 110 | 1.4 (0.92, 2.02) | 4.21 (2.99, 5.59) | 0.61 (0.45, 0.76) | 0.53 (0.31, 0.83) | 0.17 (0.11, 0.25) | 0.13 (0.07, 0.21) |
| 2013 | 102 | 1.1 (0.79, 1.67) | 2.98 (2.15, 4.4) | 0.52 (0.36, 0.75) | 0.52 (0.28, 0.91) | 0.14 (0.1, 0.22) | 0.09 (0.07, 0.19) |
| 2014 | 45 | 1.09 (0.73, 1.75) | 3.22 (2.55, 4.38) | 0.51 (0.34, 0.74) | 0.62 (0.32, 0.96) | 0.14 (0.1, 0.22) | 0.11 (0.05, 0.2) |
| 2015 | 69 | 1.18 (0.71, 1.63) | 2.85 (1.95, 4.09) | 0.47 (0.33, 0.65) | 0.54 (0.38, 0.85) | 0.15 (0.09, 0.22) | 0.13 (0.06, 0.22) |
| 2016 | 76 | 1 (0.67, 1.52) | 2.8 (1.85, 3.98) | 0.38 (0.26, 0.55) | 0.49 (0.31, 1.06) | 0.13 (0.08, 0.18) | 0.09 (0.06, 0.18) |
| 2017+ | 102 | 0.84 (0.55, 1.44) | 2.3 (1.62, 3.18) | 0.33 (0.24, 0.49) | 0.44 (0.26, 0.8) | 0.11 (0.07, 0.17) | 0.08 (0.06, 0.17) |
| **Number of ultrasound scans per participant** |  |  |  |  |  |  |  |
| 1 | 134 | 1.91 (1.21, 2.81) | 5.6 (3.55, 8.1) | 0.75 (0.51, 1.04) | 0.73 (0.41, 1.34) | 0.23 (0.16, 0.32) | 0.19 (0.12, 0.3) |
| 2 | 169 | 1.97 (1.26, 2.9) | 6.31 (3.49, 8.98) | 0.74 (0.54, 1.04) | 0.75 (0.44, 1.35) | 0.24 (0.16, 0.32) | 0.19 (0.11, 0.33) |
| 3 | 176 | 2.03 (1.06, 2.95) | 5 (3.17, 8.54) | 0.7 (0.49, 1) | 0.74 (0.38, 1.31) | 0.24 (0.14, 0.35) | 0.18 (0.09, 0.31) |
| 4 | 216 | 1.55 (0.89, 2.61) | 4.64 (2.73, 7.24) | 0.69 (0.39, 0.97) | 0.6 (0.37, 1.1) | 0.21 (0.12, 0.31) | 0.16 (0.07, 0.29) |
| 5 | 193 | 1.7 (0.92, 2.41) | 3.98 (2.68, 6.71) | 0.62 (0.42, 0.88) | 0.64 (0.33, 1.19) | 0.2 (0.12, 0.3) | 0.15 (0.07, 0.27) |
| 6 | 143 | 1.36 (0.92, 2.2) | 3.86 (2.77, 6.71) | 0.58 (0.43, 0.82) | 0.73 (0.44, 1.06) | 0.18 (0.12, 0.29) | 0.12 (0.07, 0.27) |
| 7 | 91 | 1.54 (0.91, 2.72) | 4.43 (2.82, 6.49) | 0.57 (0.38, 0.84) | 0.75 (0.43, 1.28) | 0.18 (0.1, 0.27) | 0.11 (0.06, 0.2) |
| 8 | 49 | 1.56 (0.99, 2.32) | 3.68 (2.68, 5.81) | 0.66 (0.37, 0.99) | 0.53 (0.37, 0.95) | 0.2 (0.12, 0.3) | 0.15 (0.07, 0.23) |
| 9 | 28 | 1.44 (1.11, 1.88) | 4.29 (2.76, 5.54) | 0.56 (0.41, 0.72) | 0.72 (0.47, 0.89) | 0.17 (0.11, 0.24) | 0.09 (0.07, 0.19) |
| 10 | 12 | 0.96 (0.63, 1.61) | 3.29 (1.71, 4.38) | 0.42 (0.33, 0.6) | 0.63 (0.15, 0.98) | 0.13 (0.11, 0.22) | 0.09 (0.04, 0.16) |
| 11 | 6 | 1.43 (1.24, 2.59) | 3.48 (2.9, 3.7) | 0.64 (0.49, 0.96) | 0.77 (0.53, 0.88) | 0.17 (0.14, 0.23) | 0.13 (0.11, 0.17) |
| 12 | 2 | 1.11 (0.79, 1.42) | 3.87 (3.1, 4.63) | 1.16 (1.13, 1.18) | 0.44 (0.35, 0.52) | 0.21 (0.2, 0.21) | 0.16 (0.13, 0.18) |
| 13 | 1 | 4.21 (4.21, 4.21) | 4.84 (4.84, 4.84) | 1.45 (1.45, 1.45) | 0.52 (0.52, 0.52) | 0.47 (0.47, 0.47) | 0.14 (0.14, 0.14) |
| Note: Maternal age and prepregnancy BMI were discretized to compare distributions of PFAS across different categories. | | | | | | | |

Table S2. Distributions of fetal growth measurements [median (Q1, Q3)] by demographic, behavioral, and clinical characteristics of study participants (N = 1220).

|  | **N** | **FL** | **HC** | **AC** | **EFW** | **Birthweight** |
| --- | --- | --- | --- | --- | --- | --- |
| **Maternal age (y)** |  |  |  |  |  |  |
| Above Median (> 32.8) | 557 | -0.09 (-0.77, 0.55) | 0.23 (-0.33, 0.86) | 0.11 (-0.51, 0.68) | -0.27 (-0.94, 0.41) | -0.06 (-0.76, 0.57) |
| Below Median (≤ 32.8) | 663 | -0.21 (-0.8, 0.54) | 0.13 (-0.47, 0.74) | -0.02 (-0.62, 0.59) | -0.43 (-1.06, 0.23) | -0.09 (-0.84, 0.57) |
| **Maternal Prepregnancy BMI** |  |  |  |  |  |  |
| BMI ≤ 25 | 605 | -0.22 (-0.82, 0.46) | 0.18 (-0.38, 0.75) | -0.02 (-0.6, 0.55) | -0.44 (-1.05, 0.2) | -0.16 (-0.83, 0.47) |
| 25 < BMI ≤ 30 | 313 | -0.21 (-0.83, 0.49) | 0.07 (-0.56, 0.75) | -0.02 (-0.62, 0.55) | -0.44 (-1.06, 0.22) | -0.16 (-0.93, 0.55) |
| BMI > 30 | 283 | 0.1 (-0.62, 0.72) | 0.28 (-0.33, 0.92) | 0.19 (-0.46, 0.88) | -0.13 (-0.83, 0.58) | 0.07 (-0.48, 0.78) |
| Missing | 19 | 0.45 (-0.18, 1.22) | 0.37 (-0.15, 1.34) | 0.38 (-0.23, 0.9) | 0.12 (-0.39, 0.73) | 0.22 (-0.29, 1.24) |
| **Maternal race/ethnicity** |  |  |  |  |  |  |
| African American | 179 | -0.21 (-0.86, 0.49) | -0.22 (-0.82, 0.44) | -0.26 (-0.91, 0.4) | -0.68 (-1.35, 0.02) | -0.34 (-1.11, 0.36) |
| Asian | 84 | -0.16 (-0.87, 0.48) | 0.09 (-0.57, 0.63) | -0.29 (-0.88, 0.38) | -0.55 (-1.22, -0.03) | -0.39 (-0.97, 0.27) |
| Non-Hispanic White | 693 | -0.12 (-0.77, 0.55) | 0.29 (-0.26, 0.92) | 0.15 (-0.42, 0.75) | -0.22 (-0.9, 0.44) | 0.07 (-0.67, 0.72) |
| Hispanic | 198 | -0.22 (-0.8, 0.49) | 0.13 (-0.51, 0.71) | -0.02 (-0.6, 0.58) | -0.41 (-1.09, 0.21) | -0.13 (-0.76, 0.41) |
| Other | 66 | 0.11 (-0.54, 0.66) | 0.13 (-0.33, 0.67) | -0.13 (-0.7, 0.47) | -0.41 (-0.94, 0.28) | -0.25 (-0.81, 0.29) |
| **Maternal educational attainment** |  |  |  |  |  |  |
| High school or less | 160 | -0.27 (-0.8, 0.46) | -0.13 (-0.67, 0.45) | -0.2 (-0.77, 0.4) | -0.56 (-1.24, 0.09) | -0.32 (-0.93, 0.38) |
| Some college or technical degree | 218 | -0.04 (-0.77, 0.62) | 0.15 (-0.55, 0.76) | -0.02 (-0.53, 0.55) | -0.39 (-1.06, 0.23) | -0.06 (-0.78, 0.55) |
| Bachelor's degree or more | 817 | -0.12 (-0.78, 0.54) | 0.24 (-0.31, 0.85) | 0.08 (-0.53, 0.69) | -0.3 (-0.96, 0.39) | -0.06 (-0.78, 0.6) |
| Missing | 25 | -0.15 (-0.54, 0.74) | 0.18 (-0.44, 0.86) | 0.24 (-0.48, 0.97) | -0.24 (-0.93, 0.74) | 0.24 (-0.46, 1.24) |
| **Health insurance** |  |  |  |  |  |  |
| Private insurance/HMO | 894 | -0.09 (-0.76, 0.55) | 0.24 (-0.32, 0.86) | 0.08 (-0.52, 0.68) | -0.27 (-0.95, 0.39) | -0.05 (-0.72, 0.63) |
| Self-pay or Medicaid/Mass Health | 310 | -0.28 (-0.95, 0.42) | -0.03 (-0.65, 0.56) | -0.17 (-0.72, 0.5) | -0.58 (-1.19, 0.07) | -0.19 (-0.97, 0.37) |
| Missing | 16 | 0.84 (-0.34, 1.29) | 0.21 (-0.81, 1.37) | 0.14 (-0.54, 0.98) | -0.11 (-1.01, 1.06) | 0.31 (-1.33, 1.24) |
| **Parity** |  |  |  |  |  |  |
| Parous | 696 | -0.12 (-0.78, 0.55) | 0.19 (-0.39, 0.8) | 0.06 (-0.54, 0.66) | -0.32 (-0.98, 0.36) | -0.03 (-0.68, 0.62) |
| Nulliparous | 524 | -0.17 (-0.79, 0.55) | 0.17 (-0.4, 0.78) | -0.01 (-0.6, 0.6) | -0.39 (-1.04, 0.28) | -0.26 (-0.94, 0.51) |
| **Smoking during pregnancy** |  |  |  |  |  |  |
| No | 1136 | -0.12 (-0.77, 0.55) | 0.19 (-0.39, 0.8) | 0.03 (-0.54, 0.65) | -0.35 (-1, 0.33) | -0.06 (-0.8, 0.6) |
| Yes | 83 | -0.29 (-1.07, 0.42) | -0.08 (-0.65, 0.57) | -0.13 (-0.63, 0.5) | -0.59 (-1.13, 0.11) | -0.38 (-0.89, 0.44) |
| Missing | 1 | 1.54 (1.22, 1.96) | 1.62 (1.43, 2) | 1.21 (0.84, 1.57) | 1.56 (1.21, 1.75) | 1.24 (1.24, 1.24) |
| **Alcohol during pregnancy** |  |  |  |  |  |  |
| No | 1123 | -0.12 (-0.77, 0.57) | 0.19 (-0.4, 0.81) | 0.04 (-0.54, 0.66) | -0.33 (-1, 0.35) | -0.06 (-0.77, 0.6) |
| Yes | 82 | -0.32 (-1, 0.21) | 0.04 (-0.44, 0.49) | -0.17 (-0.75, 0.48) | -0.6 (-1.19, -0.04) | -0.41 (-1.08, 0.29) |
| Missing | 15 | 0.16 (-0.46, 1.16) | 0.29 (-0.35, 1.28) | 0.56 (-0.2, 1.18) | -0.12 (-0.62, 0.88) | 0.75 (-1.33, 1.24) |
| **Fetal sex** |  |  |  |  |  |  |
| Female | 567 | -0.22 (-0.83, 0.48) | 0 (-0.58, 0.54) | -0.06 (-0.6, 0.5) | -0.46 (-1.08, 0.15) | -0.27 (-0.88, 0.34) |
| Male | 649 | -0.06 (-0.73, 0.58) | 0.36 (-0.24, 0.98) | 0.14 (-0.53, 0.74) | -0.24 (-0.95, 0.47) | 0.09 (-0.72, 0.75) |
| Missing | 4 | -0.13 (-0.51, 0.21) | -0.1 (-0.27, 0.42) | -0.31 (-0.9, 0.43) | -0.71 (-1.05, 0.04) | -0.9 (-0.9, -0.9) |
| **Year of sample collection** |  |  |  |  |  |  |
| 2006 | 47 | -0.21 (-0.97, 0.48) | 0.22 (-0.56, 0.67) | -0.02 (-0.67, 0.58) | -0.48 (-1.09, 0.33) | -0.11 (-1.01, 0.86) |
| 2007 | 264 | 0.17 (-0.52, 0.82) | 0.21 (-0.38, 0.83) | 0.08 (-0.54, 0.8) | -0.24 (-0.96, 0.49) | 0.11 (-0.77, 0.76) |
| 2008 | 203 | 0.04 (-0.63, 0.72) | 0.25 (-0.42, 0.88) | 0.02 (-0.63, 0.56) | -0.31 (-1.04, 0.33) | -0.09 (-0.78, 0.67) |
| 2009 | 34 | -0.14 (-0.81, 0.77) | 0.26 (-0.38, 0.97) | 0.02 (-0.59, 0.9) | -0.46 (-1.02, 0.37) | -0.51 (-0.89, 0.56) |
| 2010 | 59 | -0.21 (-0.8, 0.49) | 0.19 (-0.28, 0.81) | 0.04 (-0.56, 0.59) | -0.33 (-0.99, 0.45) | -0.06 (-0.58, 0.5) |
| 2011 | 109 | -0.28 (-1.01, 0.36) | 0.1 (-0.44, 0.78) | -0.07 (-0.57, 0.44) | -0.51 (-1.04, 0.16) | -0.28 (-0.83, 0.36) |
| 2012 | 110 | -0.26 (-0.85, 0.44) | 0.07 (-0.49, 0.54) | -0.07 (-0.66, 0.52) | -0.48 (-1.11, 0.19) | -0.25 (-0.93, 0.45) |
| 2013 | 102 | -0.23 (-0.78, 0.37) | 0.08 (-0.51, 0.65) | -0.06 (-0.58, 0.52) | -0.48 (-1.02, 0.2) | -0.09 (-0.89, 0.55) |
| 2014 | 45 | -0.26 (-0.83, 0.22) | 0.23 (-0.29, 0.78) | 0.14 (-0.4, 0.74) | -0.37 (-0.9, 0.21) | -0.25 (-0.72, 0.32) |
| 2015 | 69 | -0.33 (-1.04, 0.4) | 0.13 (-0.61, 0.8) | -0.15 (-0.87, 0.5) | -0.54 (-1.18, 0.24) | -0.35 (-0.95, 0.23) |
| 2016 | 76 | -0.29 (-0.96, 0.21) | 0.17 (-0.38, 0.77) | 0.15 (-0.43, 0.79) | -0.24 (-0.85, 0.37) | 0.02 (-0.57, 0.62) |
| 2017+ | 102 | -0.05 (-0.78, 0.59) | 0.4 (-0.11, 1.02) | 0.24 (-0.33, 0.76) | -0.08 (-0.77, 0.53) | 0.13 (-0.49, 0.61) |
| **Preterm birth** |  |  |  |  |  |  |
| No | 865 | -0.12 (-0.77, 0.58) | 0.19 (-0.38, 0.78) | 0.02 (-0.54, 0.6) | -0.33 (-0.98, 0.33) | -0.14 (-0.82, 0.55) |
| Yes | 355 | -0.21 (-0.83, 0.46) | 0.16 (-0.44, 0.82) | 0.04 (-0.62, 0.69) | -0.39 (-1.08, 0.31) | 0.09 (-0.68, 0.64) |
| **Categorized birthweight status** |  |  |  |  |  |  |
| SGA | 116 | -0.74 (-1.48, -0.13) | -0.46 (-1.11, 0.05) | -0.94 (-1.52, -0.41) | -1.25 (-1.8, -0.78) | -1.64 (-1.9, -1.4) |
| AGA | 982 | -0.12 (-0.77, 0.54) | 0.2 (-0.36, 0.79) | 0.04 (-0.49, 0.59) | -0.32 (-0.93, 0.28) | -0.07 (-0.6, 0.43) |
| LGA | 114 | 0.49 (-0.12, 1.06) | 0.83 (0.27, 1.44) | 0.99 (0.41, 1.59) | 0.67 (-0.11, 1.55) | 1.74 (1.47, 2.27) |
| Missing | 8 | -0.22 (-0.77, 0.41) | 0.3 (-0.15, 0.73) | 0.23 (-0.48, 0.78) | -0.29 (-0.7, 0.26) | NA |
| **Number of ultrasound scans per participant** | |  |  |  |  |  |
| 1 | 134 | 0.2 (-0.55, 0.72) | 0.25 (-0.32, 1.03) | 0.2 (-0.54, 0.82) | -0.78 (-1.41, -0.01) | 0.08 (-0.75, 0.62) |
| 2 | 169 | 0.19 (-0.42, 0.8) | 0.37 (-0.17, 1) | 0.08 (-0.41, 0.75) | -0.37 (-0.94, 0.27) | -0.03 (-0.54, 0.55) |
| 3 | 176 | 0.09 (-0.73, 0.72) | 0.24 (-0.39, 0.88) | 0.15 (-0.46, 0.71) | -0.29 (-0.93, 0.43) | 0.06 (-0.53, 0.77) |
| 4 | 216 | -0.04 (-0.65, 0.58) | 0.27 (-0.3, 0.84) | 0.14 (-0.43, 0.74) | -0.21 (-0.91, 0.4) | 0 (-0.65, 0.73) |
| 5 | 193 | -0.21 (-0.82, 0.55) | 0.2 (-0.35, 0.77) | 0 (-0.55, 0.54) | -0.34 (-0.95, 0.27) | -0.13 (-0.83, 0.47) |
| 6 | 143 | -0.22 (-0.82, 0.46) | 0.08 (-0.51, 0.72) | 0.03 (-0.53, 0.68) | -0.42 (-0.99, 0.35) | -0.15 (-0.74, 0.5) |
| 7 | 91 | -0.03 (-0.65, 0.6) | 0.23 (-0.33, 0.85) | 0.04 (-0.54, 0.65) | -0.24 (-0.9, 0.39) | -0.13 (-0.9, 0.62) |
| 8 | 49 | -0.28 (-0.84, 0.29) | 0.11 (-0.46, 0.8) | 0.03 (-0.61, 0.64) | -0.38 (-1.06, 0.3) | -0.19 (-1.02, 0.53) |
| 9 | 28 | -0.5 (-1.32, 0.28) | 0.06 (-0.56, 0.63) | -0.27 (-0.76, 0.38) | -0.65 (-1.22, 0.12) | -0.11 (-0.93, 0.48) |
| 10 | 12 | -0.54 (-1.21, 0.18) | -0.23 (-0.97, 0.38) | -0.34 (-1.08, 0.42) | -0.59 (-1.59, 0.11) | -0.52 (-1.11, 0.27) |
| 11 | 6 | -0.74 (-1.11, -0.11) | -0.03 (-0.54, 0.72) | -0.46 (-1.29, 0.44) | -0.9 (-1.48, -0.28) | -0.92 (-1.4, -0.51) |
| 12 | 2 | -1.99 (-2.88, -0.81) | -0.21 (-1.04, -0.02) | -0.51 (-1.85, 0.48) | -1.41 (-2.43, -0.36) | -1.15 (-2.59, 0.29) |
| 13 | 1 | -0.17 (-0.86, 0.57) | -0.79 (-1.41, 0.06) | -1.33 (-1.65, -1.01) | -1.35 (-1.96, -0.93) | -0.35 (-0.35, -0.35) |
| Note: Maternal age and prepregnancy BMI were discretized to compare distributions of fetal growth measurements across different categories. The distributions are based on the Z-scored fetal growth measurements. | | | | | | |

Table S3. Distributions of mean gestational age (GA) by scan number.

| Scan number | Mean gestational age | Number of participants |
| --- | --- | --- |
| 1 | 17.89 | 1220 |
| 2 | 24.62 | 1086 |
| 3 | 28.46 | 917 |
| 4 | 30.83 | 741 |
| 5 | 32.35 | 525 |
| 6 | 33.13 | 332 |
| 7 | 33.78 | 189 |
| 8 | 34.01 | 98 |
| 9 | 34.55 | 49 |
| 10 | 34.57 | 21 |
| 11 | 34.89 | 9 |
| 12 | 34.00 | 3 |
| 13 | 30.00 | 1 |

Table S4. Associations between prenatal PFAS exposure and ultrasound measures of fetal growth.

| PFAS | Group | FL | | HC | | AC | | EFW | |
| --- | --- | --- | --- | --- | --- | --- | --- | --- | --- |
|  |  | Estimate (95% CI) | p-value | Estimate (95% CI) | p-value | Estimate (95% CI) | p-value | Estimate (95% CI) | p-value |
| PFOA | All | 0.1 (0.02, 0.18) | 0.02 | -0.01 (-0.09, 0.06) | 0.69 | -0.02 (-0.1, 0.06) | 0.61 | 0.01 (-0.07, 0.09) | 0.81 |
|  | Male | 0.16 (0.05, 0.28) | 0.01 | 0.04 (-0.05, 0.14) | 0.37 | -0.01 (-0.11, 0.09) | 0.86 | 0.05 (-0.06, 0.16) | 0.37 |
|  | Female | 0 (-0.12, 0.13) | 0.95 | -0.09 (-0.19, 0.01) | 0.08 | -0.04 (-0.16, 0.07) | 0.48 | -0.05 (-0.17, 0.07) | 0.4 |
|  |  |  |  |  |  |  |  |  |  |
| PFOS | All | 0.08 (0, 0.15) | 0.06 | 0 (-0.06, 0.07) | 0.94 | 0.03 (-0.05, 0.1) | 0.47 | 0.03 (-0.04, 0.11) | 0.38 |
|  | Male | 0.08 (-0.03, 0.19) | 0.16 | 0.04 (-0.05, 0.14) | 0.38 | 0.04 (-0.07, 0.15) | 0.49 | 0.05 (-0.06, 0.16) | 0.34 |
|  | Female | 0.06 (-0.05, 0.17) | 0.28 | -0.04 (-0.14, 0.05) | 0.38 | 0.01 (-0.08, 0.11) | 0.8 | 0 (-0.1, 0.11) | 0.95 |
|  |  |  |  |  |  |  |  |  |  |
| PFNA | All | 0.05 (-0.02, 0.12) | 0.14 | 0.01 (-0.05, 0.06) | 0.8 | -0.04 (-0.1, 0.02) | 0.22 | -0.01 (-0.07, 0.06) | 0.85 |
|  | Male | 0.06 (-0.03, 0.14) | 0.21 | 0.03 (-0.05, 0.1) | 0.48 | -0.03 (-0.11, 0.05) | 0.4 | 0.01 (-0.08, 0.09) | 0.89 |
|  | Female | 0.03 (-0.07, 0.13) | 0.55 | -0.01 (-0.09, 0.06) | 0.73 | -0.05 (-0.13, 0.04) | 0.31 | -0.03 (-0.12, 0.06) | 0.53 |
|  |  |  |  |  |  |  |  |  |  |
| PFHxS | All | 0.06 (-0.01, 0.12) | 0.08 | 0 (-0.06, 0.05) | 0.89 | 0.02 (-0.04, 0.08) | 0.56 | 0.01 (-0.05, 0.08) | 0.7 |
|  | Male | 0.05 (-0.03, 0.14) | 0.22 | -0.02 (-0.1, 0.05) | 0.56 | 0.01 (-0.07, 0.1) | 0.78 | 0.01 (-0.08, 0.1) | 0.84 |
|  | Female | 0.04 (-0.05, 0.13) | 0.34 | 0.01 (-0.06, 0.09) | 0.73 | 0.02 (-0.07, 0.11) | 0.65 | 0 (-0.08, 0.09) | 0.96 |
|  |  |  |  |  |  |  |  |  |  |
| PFDA | All | 0.05 (-0.02, 0.11) | 0.16 | 0.02 (-0.03, 0.07) | 0.46 | -0.01 (-0.07, 0.06) | 0.87 | 0.02 (-0.04, 0.09) | 0.53 |
|  | Male | 0.05 (-0.04, 0.13) | 0.27 | 0.04 (-0.02, 0.11) | 0.19 | 0 (-0.08, 0.07) | 0.92 | 0.03 (-0.05, 0.11) | 0.44 |
|  | Female | 0.05 (-0.06, 0.15) | 0.37 | -0.02 (-0.1, 0.06) | 0.69 | -0.01 (-0.11, 0.09) | 0.89 | 0 (-0.1, 0.11) | 0.98 |
|  |  |  |  |  |  |  |  |  |  |
| PFUA | All | 0.05 (-0.02, 0.12) | 0.19 | 0.02 (-0.04, 0.09) | 0.49 | -0.04 (-0.11, 0.02) | 0.19 | 0 (-0.07, 0.07) | 0.96 |
|  | Male | 0.09 (-0.01, 0.19) | 0.09 | 0.07 (-0.03, 0.17) | 0.16 | 0 (-0.11, 0.1) | 0.96 | 0.05 (-0.06, 0.16) | 0.38 |
|  | Female | 0 (-0.1, 0.09) | 0.97 | -0.03 (-0.11, 0.06) | 0.53 | -0.09 (-0.18, 0) | 0.04 | -0.06 (-0.15, 0.03) | 0.19 |
| Note: Estimates derived from generalized estimating equations with exchangeable correlation. Effect estimates (95% confidence intervals) show the change in standard deviations of each measure per interquartile range increase in log-transformed PFAS concentrations. All models were adjusted for maternal age, prepregnancy BMI, race/ethnicity, education, insurance status, parity, enrollment year, and number of ultrasound scans, with additional adjustment for fetal sex in the “All” group. Separate models were run for each fetal sex. The sample size (number of records, participants) for each measure was: FL: All (4980, 1173), male (2625, 625), and female (2355, 548); HC: All (4825, 1126), male (2545, 598), and female (2280, 528); AC: All (4854, 1126), male (2558, 598), and female (2296, 528); EFW: All (4847, 1126), male (2553, 598), and female (2294, 528). | | | | | | | | | |

Table S5. P-values for the interaction term related to fetal sex.

| **PFAS** | **FL** | **HC** | **AC** | **EFW** | **Birthweight** | **LGA** | **SGA** |  |
| --- | --- | --- | --- | --- | --- | --- | --- | --- |
| PFOA | 0.45 | 0.59 | 0.72 | 0.78 | 0.11 | <0.01 | 0.26 |  |
| PFOS | 0.04 | 0.97 | 0.76 | 0.46 | 0.07 | <0.01 | 0.79 |  |
| PFNA | 0.13 | 0.82 | 0.72 | 0.59 | <0.01 | <0.01 | 0.03 |  |
| PFHxS | 0.22 | 0.26 | 0.59 | 0.51 | 0.11 | <0.01 | 0.70 |  |
| PFDA | 0.11 | 0.85 | 0.74 | 0.68 | <0.01 | <0.01 | 0.02 |  |
| PFUA | 0.92 | 0.52 | 0.39 | 0.44 | 0.13 | 0.01 | 0.09 |  |
| Note: P-values correspond to PFAS x fetal sex interaction terms from generalized estimating equation (GEE) models (for FL, HC, and AC), linear regression models (BW), and logistic regression models (LGA, SGA). All models were adjusted for maternal age, prepregnancy BMI, race/ethnicity, education, insurance status, parity, enrollment year, and number of ultrasound scans. | | | | | | | |  |
|  |  |  |  |  |  |  |  |  |

Table S6. Associations between prenatal PFAS exposure and ultrasound measures of fetal growth stratified by maternal race.

| PFAS | Group | FL | | HC | | AC | | EFW | |
| --- | --- | --- | --- | --- | --- | --- | --- | --- | --- |
|  |  | Estimate (95% CI) | p-value | Estimate (95% CI) | p-value | Estimate (95% CI) | p-value | Estimate (95% CI) | p-value |
| PFOA | Non-Hispanic White | 0.1 (-0.02, 0.22) | 0.09 | -0.04 (-0.14, 0.06) | 0.44 | -0.04 (-0.14, 0.05) | 0.37 | -0.02 (-0.14, 0.09) | 0.7 |
|  | African American | 0.26 (-0.01, 0.53) | 0.06 | 0.1 (-0.13, 0.33) | 0.38 | 0.18 (-0.05, 0.41) | 0.13 | 0.2 (-0.04, 0.45) | 0.1 |
|  | Hispanic | -0.05 (-0.2, 0.1) | 0.51 | -0.07 (-0.22, 0.08) | 0.37 | -0.08 (-0.25, 0.08) | 0.33 | -0.09 (-0.24, 0.07) | 0.27 |
|  | Asian/Other | 0.16 (-0.06, 0.37) | 0.16 | 0.08 (-0.11, 0.27) | 0.4 | -0.04 (-0.27, 0.18) | 0.71 | 0.06 (-0.16, 0.28) | 0.59 |
|  |  |  |  |  |  |  |  |  |  |
| PFOS | Non-Hispanic White | 0.08 (-0.03, 0.19) | 0.15 | 0 (-0.1, 0.1) | 0.99 | 0.03 (-0.08, 0.13) | 0.62 | 0.02 (-0.09, 0.13) | 0.76 |
|  | African American | 0.21 (0, 0.42) | 0.05 | 0.02 (-0.18, 0.22) | 0.84 | 0.13 (-0.1, 0.35) | 0.27 | 0.16 (-0.06, 0.38) | 0.16 |
|  | Hispanic | 0.03 (-0.13, 0.19) | 0.71 | 0 (-0.13, 0.14) | 0.95 | 0.02 (-0.11, 0.15) | 0.75 | 0.01 (-0.13, 0.15) | 0.89 |
|  | Asian/Other | 0.04 (-0.13, 0.21) | 0.61 | 0.03 (-0.15, 0.21) | 0.73 | -0.02 (-0.2, 0.15) | 0.79 | 0.02 (-0.15, 0.2) | 0.79 |
|  |  |  |  |  |  |  |  |  |  |
| PFNA | Non-Hispanic White | 0.04 (-0.05, 0.14) | 0.35 | 0.01 (-0.06, 0.08) | 0.79 | -0.05 (-0.13, 0.03) | 0.21 | -0.02 (-0.1, 0.07) | 0.67 |
|  | African American | 0.19 (0.01, 0.38) | 0.04 | -0.01 (-0.21, 0.18) | 0.9 | 0.03 (-0.18, 0.25) | 0.76 | 0.06 (-0.15, 0.26) | 0.57 |
|  | Hispanic | 0.05 (-0.07, 0.18) | 0.42 | -0.01 (-0.13, 0.1) | 0.8 | -0.06 (-0.18, 0.06) | 0.32 | -0.03 (-0.15, 0.09) | 0.58 |
|  | Asian/Other | -0.02 (-0.2, 0.15) | 0.78 | 0.09 (-0.06, 0.23) | 0.25 | -0.04 (-0.21, 0.13) | 0.63 | 0.02 (-0.16, 0.19) | 0.85 |
|  |  |  |  |  |  |  |  |  |  |
| PFHxS | Non-Hispanic White | 0.02 (-0.06, 0.11) | 0.6 | -0.07 (-0.14, 0.01) | 0.1 | -0.01 (-0.09, 0.07) | 0.77 | -0.04 (-0.12, 0.05) | 0.43 |
|  | African American | 0.2 (0.01, 0.38) | 0.04 | 0.11 (-0.06, 0.29) | 0.21 | 0.05 (-0.16, 0.26) | 0.65 | 0.12 (-0.09, 0.32) | 0.26 |
|  | Hispanic | 0.05 (-0.08, 0.18) | 0.44 | 0.07 (-0.03, 0.17) | 0.17 | 0.04 (-0.07, 0.15) | 0.47 | 0.03 (-0.08, 0.14) | 0.6 |
|  | Asian/Other | 0.11 (-0.06, 0.28) | 0.19 | 0.1 (-0.03, 0.22) | 0.12 | 0.1 (-0.08, 0.28) | 0.29 | 0.12 (-0.05, 0.28) | 0.16 |
|  |  |  |  |  |  |  |  |  |  |
| PFDA | Non-Hispanic White | 0.09 (-0.01, 0.18) | 0.08 | 0.03 (-0.05, 0.11) | 0.49 | 0.02 (-0.07, 0.12) | 0.67 | 0.06 (-0.04, 0.16) | 0.24 |
|  | African American | 0.13 (-0.06, 0.32) | 0.19 | 0.03 (-0.14, 0.2) | 0.76 | -0.01 (-0.2, 0.17) | 0.89 | 0 (-0.19, 0.19) | 0.97 |
|  | Hispanic | 0.04 (-0.07, 0.15) | 0.48 | -0.01 (-0.1, 0.08) | 0.81 | -0.01 (-0.11, 0.08) | 0.77 | -0.01 (-0.11, 0.09) | 0.9 |
|  | Asian/Other | -0.07 (-0.19, 0.06) | 0.29 | 0.05 (-0.06, 0.15) | 0.37 | -0.04 (-0.15, 0.08) | 0.51 | -0.02 (-0.14, 0.1) | 0.73 |
|  |  |  |  |  |  |  |  |  |  |
| PFUA | Non-Hispanic White | 0.07 (-0.03, 0.17) | 0.18 | 0.07 (-0.02, 0.15) | 0.14 | -0.04 (-0.13, 0.05) | 0.37 | 0.01 (-0.08, 0.11) | 0.77 |
|  | African American | 0.05 (-0.16, 0.25) | 0.66 | -0.23 (-0.44, -0.03) | 0.03 | -0.13 (-0.33, 0.07) | 0.21 | -0.15 (-0.34, 0.04) | 0.13 |
|  | Hispanic | 0.07 (-0.09, 0.24) | 0.39 | -0.05 (-0.2, 0.09) | 0.45 | -0.08 (-0.23, 0.07) | 0.3 | -0.04 (-0.2, 0.11) | 0.57 |
|  | Asian/Other | -0.05 (-0.21, 0.12) | 0.58 | 0.03 (-0.16, 0.21) | 0.77 | -0.02 (-0.18, 0.14) | 0.79 | 0.02 (-0.15, 0.19) | 0.84 |
| Note: Estimates derived from generalized estimating equations with exchangeable correlation. Effect estimates (95% confidence intervals) show the change in standard deviations of each measure per interquartile range increase in log-transformed PFAS concentrations. All models were adjusted for maternal age, prepregnancy BMI, education, insurance status, parity, fetal sex, enrollment year, and number of ultrasound scans. The sample size (number of records, participants) for each measure was: FL: Non-Hispanic White (2965, 672), African American (693, 169), Hispanic (750, 188), and Asian/Other (572, 144); HC: Non-Hispanic White (2849, 633), African American (675, 165), Hispanic (749, 188), and Asian/Other (552, 140); AC: Non-Hispanic White (2862, 633), African American (681, 165), Hispanic (750, 188), and Asian/Other (561, 140); EFW: Non-Hispanic White (2860, 633), African American (680, 165), Hispanic (748, 188), and Asian/Other (559, 140). | | | | | | | | | |

Table S7. Associations between prenatal PFAS exposure and birthweight Z-scores among all participants and across different subsets.

| PFAS | Group | N | Estimate (95% CI) | p-value |
| --- | --- | --- | --- | --- |
| PFOA | All | 1168 | -0.01(-0.11, 0.09) | 0.856 |
|  | Male | 621 | -0.07(-0.21, 0.06) | 0.284 |
|  | Female | 547 | 0.03(-0.13, 0.18) | 0.716 |
|  | Non-Hispanic White | 670 | -0.07(-0.22, 0.08) | 0.353 |
|  | African American | 168 | 0.44(0.15, 0.73) | 0.003 |
|  | Hispanic | 186 | -0.21(-0.43, 0) | 0.056 |
|  | Asian/Other | 144 | 0.13(-0.11, 0.37) | 0.289 |
|  |  |  |  |  |
| PFOS | All | 1168 | 0.03(-0.07, 0.13) | 0.524 |
|  | Male | 621 | -0.03(-0.17, 0.11) | 0.66 |
|  | Female | 547 | 0.07(-0.07, 0.21) | 0.352 |
|  | Non-Hispanic White | 670 | 0.03(-0.11, 0.17) | 0.668 |
|  | African American | 168 | 0.23(-0.04, 0.5) | 0.094 |
|  | Hispanic | 186 | -0.17(-0.42, 0.07) | 0.171 |
|  | Asian/Other | 144 | 0.09(-0.11, 0.28) | 0.392 |
|  |  |  |  |  |
| PFNA | All | 1168 | 0(-0.08, 0.08) | 0.969 |
|  | Male | 621 | -0.09(-0.21, 0.02) | 0.1 |
|  | Female | 547 | 0.09(-0.03, 0.21) | 0.132 |
|  | Non-Hispanic White | 670 | -0.03(-0.15, 0.08) | 0.581 |
|  | African American | 168 | 0.23(0, 0.46) | 0.053 |
|  | Hispanic | 186 | -0.13(-0.32, 0.05) | 0.162 |
|  | Asian/Other | 144 | 0.16(-0.03, 0.35) | 0.094 |
|  |  |  |  |  |
| PFHxS | All | 1168 | -0.01(-0.1, 0.07) | 0.763 |
|  | Male | 621 | -0.08(-0.19, 0.03) | 0.143 |
|  | Female | 547 | 0.05(-0.07, 0.18) | 0.427 |
|  | Non-Hispanic White | 670 | -0.06(-0.17, 0.05) | 0.306 |
|  | African American | 168 | 0.22(-0.02, 0.46) | 0.074 |
|  | Hispanic | 186 | -0.13(-0.32, 0.07) | 0.201 |
|  | Asian/Other | 144 | 0.1(-0.08, 0.28) | 0.287 |
|  |  |  |  |  |
| PFDA | All | 1168 | 0.04(-0.04, 0.11) | 0.31 |
|  | Male | 621 | -0.04(-0.13, 0.05) | 0.399 |
|  | Female | 547 | 0.15(0.03, 0.27) | 0.013 |
|  | Non-Hispanic White | 670 | 0.06(-0.05, 0.18) | 0.297 |
|  | African American | 168 | 0.17(-0.05, 0.39) | 0.123 |
|  | Hispanic | 186 | -0.09(-0.25, 0.06) | 0.239 |
|  |  |  |  |  |
|  | Asian/Other | 144 | 0.09(-0.05, 0.23) | 0.223 |
|  |  |  |  |  |
| PFUA | All | 1168 | 0.01(-0.08, 0.11) | 0.753 |
|  | Male | 621 | -0.03(-0.16, 0.1) | 0.629 |
|  | Female | 547 | 0.05(-0.08, 0.18) | 0.464 |
|  | Non-Hispanic White | 670 | 0.04(-0.09, 0.16) | 0.585 |
|  | African American | 168 | -0.01(-0.3, 0.27) | 0.93 |
|  | Hispanic | 186 | -0.13(-0.37, 0.11) | 0.276 |
|  | Asian/Other | 144 | 0.1(-0.08, 0.27) | 0.283 |
| Note: Associations between prenatal PFAS exposure and standardized birthweight measurements estimated using linear regression models. Coefficients (95% CI) represent changes in birthweight Z-score per interquartile range increase in log‐transformed PFAS concentrations. Models for the entire cohort were adjusted for maternal age, prepregnancy BMI, race/ethnicity, education, insurance status, parity, enrollment year, and fetal sex. Fetal sex and maternal race-stratified models were not adjusted for fetal sex and maternal race, respectively. | | | | |

Table S8. Associations between prenatal PFAS exposure and categorized measures of extreme birthweight Z-scores.

|  | | LGA | | | | SGA | | | |
| --- | --- | --- | --- | --- | --- | --- | --- | --- | --- |
| PFAS | Group | N | events | OR (95% CI) | p-value | N | events | OR (95% CI) | p-value |
| PFOA | All | 1057 | 108 | 0.97(0.8, 1.19) | 0.8 | 1060 | 111 | 0.89(0.74, 1.08) | 0.25 |
|  | Male | 568 | 69 | 0.77(0.6, 0.99) | 0.04 | 552 | 53 | 0.95(0.7, 1.29) | 0.74 |
|  | Female | 489 | 39 | 1.48(1.02, 2.14) | 0.04 | 508 | 58 | 0.84(0.64, 1.09) | 0.2 |
|  |  |  |  |  |  |  |  |  |  |
| PFOS | All | 1057 | 108 | 0.82(0.67, 0.99) | 0.04 | 1060 | 111 | 0.72(0.61, 0.86) | 0 |
|  | Male | 568 | 69 | 0.63(0.49, 0.81) | 0 | 552 | 53 | 0.61(0.47, 0.79) | 0 |
|  | Female | 489 | 39 | 1.13(0.83, 1.56) | 0.44 | 508 | 58 | 0.8(0.63, 1.01) | 0.06 |
|  |  |  |  |  |  |  |  |  |  |
| PFNA | All | 1057 | 108 | 0.85(0.72, 0.99) | 0.04 | 1060 | 111 | 0.96(0.83, 1.12) | 0.62 |
|  | Male | 568 | 69 | 0.75(0.61, 0.92) | 0.01 | 552 | 53 | 1.12(0.88, 1.42) | 0.36 |
|  | Female | 489 | 39 | 1.05(0.79, 1.39) | 0.76 | 508 | 58 | 0.86(0.7, 1.06) | 0.15 |
|  |  |  |  |  |  |  |  |  |  |
| PFHxS | All | 1057 | 108 | 0.9(0.77, 1.05) | 0.19 | 1060 | 111 | 0.75(0.65, 0.88) | 0 |
|  | Male | 568 | 69 | 0.7(0.57, 0.85) | 0 | 552 | 53 | 0.72(0.58, 0.9) | 0 |
|  | Female | 489 | 39 | 1.37(1.04, 1.81) | 0.02 | 508 | 58 | 0.74(0.6, 0.93) | 0.01 |
|  |  |  |  |  |  |  |  |  |  |
| PFDA | All | 1057 | 108 | 1(0.85, 1.16) | 0.96 | 1060 | 111 | 0.89(0.79, 1.02) | 0.09 |
|  | Male | 568 | 69 | 0.83(0.69, 1) | 0.05 | 552 | 53 | 0.99(0.83, 1.2) | 0.95 |
|  | Female | 489 | 39 | 1.55(1.15, 2.11) | 0 | 508 | 58 | 0.77(0.64, 0.94) | 0.01 |
|  |  |  |  |  |  |  |  |  |  |
| PFUA | All | 1057 | 108 | 0.97(0.81, 1.16) | 0.72 | 1060 | 111 | 0.94(0.79, 1.12) | 0.49 |
|  | Male | 568 | 69 | 0.86(0.69, 1.09) | 0.21 | 552 | 53 | 1.04(0.79, 1.38) | 0.77 |
|  | Female | 489 | 39 | 1.19(0.87, 1.62) | 0.28 | 508 | 58 | 0.86(0.69, 1.08) | 0.19 |
| Note: Associations between prenatal PFAS exposure and large-for-gestational-age (LGA) and small-for-gestational-age (SGA) outcomes were estimated using logistic regression models. Odds ratios (OR) and 95% CIs represent the odds of SGA or LGA per interquartile range increase in log-transformed PFAS concentrations. Models for the entire cohort were adjusted for maternal age, prepregnancy BMI, race/ethnicity, education, insurance status, parity, enrollment year, and fetal sex. Models stratified by fetal sex (Male and Female) were not adjusted for fetal sex. | | | | | | | | | |

Table S9. Associations between prenatal PFAS mixture and ultrasound measures of fetal growth among all participants and across different subsets.

| Group | FL | | HC | | AC | | EFW | |
| --- | --- | --- | --- | --- | --- | --- | --- | --- |
|  | Estimate (95% CI) | p-value | Estimate (95% CI) | p-value | Estimate (95% CI) | p-value | Estimate (95% CI) | p-value |
| All | 0.11 (0.04, 0.19) | 0.004 | 0 (-0.07, 0.07) | 0.948 | 0 (-0.07, 0.08) | 0.921 | 0.04 (-0.04, 0.12) | 0.341 |
| Male | 0.12 (0.02, 0.22) | 0.022 | 0.02 (-0.07, 0.11) | 0.637 | 0.02 (-0.07, 0.12) | 0.624 | 0.06 (-0.04, 0.16) | 0.212 |
| Female | 0.09 (-0.02, 0.21) | 0.106 | -0.03 (-0.14, 0.07) | 0.545 | -0.02 (-0.12, 0.09) | 0.713 | 0.01 (-0.11, 0.12) | 0.921 |
| Note: Associations between a PFAS mixture (PFOA, PFOS, PFNA, PFHxS, PFDA and PFUA) and standardized ultrasound measures of fetal growth. Estimates derived from quantile-based g-computation (qgcomp) using 1000 Monte Carlo simulations and 5000 bootstrap samples under a linear model to obtain robust standard errors. Effect estimates (95% confidence intervals) show the change in standard deviations of each growth measure per quartile increase in PFAS levels. Models for the entire cohort were adjusted for maternal age, prepregnancy BMI, race/ethnicity, education, insurance status, parity, fetal sex, enrollment year and number of ultrasound scans. | | | | | | | | |

Table S10. Associations between prenatal PFAS mixture and birthweight Z-scores among all participants and across different subsets.

| Group | Sample size | Estimate (95% CI) | p-value |
| --- | --- | --- | --- |
| All | 1168 | 0.03 (-0.06, 0.12) | 0.5 |
| Male | 621 | -0.05 (-0.17, 0.07) | 0.39 |
| Female | 547 | 0.10 (-0.03, 0.23) | 0.12 |
| Note: Associations between a PFAS mixture (PFOA, PFOS, PFNA, PFHxS, PFDA and PFUA) and birthweight Z-score. Estimates derived from quantile based g‑computation (qgcomp) under a linear model assumption. Effect estimates (95% confidence intervals) show the change in birthweight Z-score per quartile increase in PFAS levels. All models were adjusted for maternal age, prepregnancy BMI, race/ethnicity, education, insurance status, parity, and enrollment year. Model for the entire cohort was also adjusted for fetal sex. | | | |

Table S11. Sensitivity analyses related to the associations between prenatal PFAS exposure and ultrasound measures of fetal growth.

| Sensitivity analysis | PFAS | FL | | HC | | AC | | EFW | |
| --- | --- | --- | --- | --- | --- | --- | --- | --- | --- |
|  |  | Estimate (95% CI) | p-value | Estimate (95% CI) | p-value | Estimate (95% CI) | p-value | Estimate (95% CI) | p-value |
| Using raw values | PFOA | 0.23 (0, 0.46) | 0.05 | -0.37 (-1.13, 0.4) | 0.35 | -0.38 (-1.57, 0.8) | 0.52 | -2.51 (-21.64, 16.61) | 0.8 |
|  | PFOS | 0.18 (-0.03, 0.4) | 0.1 | -0.14 (-0.86, 0.58) | 0.7 | 0.35 (-0.77, 1.47) | 0.54 | 3.66 (-12.66, 19.99) | 0.66 |
|  | PFNA | 0.12 (-0.06, 0.3) | 0.2 | -0.01 (-0.6, 0.57) | 0.97 | -0.48 (-1.39, 0.42) | 0.3 | -1.4 (-15.22, 12.42) | 0.84 |
|  | PFHxS | 0.13 (-0.04, 0.31) | 0.13 | -0.19 (-0.78, 0.39) | 0.51 | 0.31 (-0.64, 1.26) | 0.52 | 4.12 (-10.04, 18.29) | 0.57 |
|  | PFDA | 0.12 (-0.07, 0.3) | 0.21 | 0.14 (-0.41, 0.69) | 0.61 | -0.01 (-0.94, 0.91) | 0.98 | 4.24 (-9.56, 18.04) | 0.55 |
|  | PFUA | 0.11 (-0.08, 0.3) | 0.26 | 0.16 (-0.54, 0.85) | 0.66 | -0.65 (-1.65, 0.34) | 0.2 | -4.29 (-18.79, 10.22) | 0.56 |
|  |  |  |  |  |  |  |  |  |  |
| Excluding smokers | PFOA | 0.11 (0.02, 0.19) | 0.02 | -0.01 (-0.09, 0.06) | 0.69 | -0.02 (-0.09, 0.06) | 0.7 | 0.02 (-0.07, 0.1) | 0.71 |
|  | PFOS | 0.08 (0, 0.16) | 0.05 | 0.01 (-0.06, 0.08) | 0.83 | 0.05 (-0.03, 0.12) | 0.21 | 0.05 (-0.03, 0.13) | 0.2 |
|  | PFNA | 0.05 (-0.02, 0.12) | 0.15 | 0.01 (-0.04, 0.07) | 0.67 | -0.02 (-0.09, 0.04) | 0.44 | 0.01 (-0.06, 0.07) | 0.87 |
|  | PFHxS | 0.06 (0, 0.13) | 0.05 | 0 (-0.06, 0.05) | 0.88 | 0.03 (-0.04, 0.09) | 0.41 | 0.02 (-0.04, 0.09) | 0.5 |
|  | PFDA | 0.04 (-0.03, 0.11) | 0.26 | 0.02 (-0.03, 0.07) | 0.42 | 0 (-0.06, 0.06) | 0.97 | 0.02 (-0.04, 0.09) | 0.48 |
|  | PFUA | 0.05 (-0.03, 0.12) | 0.2 | 0.04 (-0.03, 0.11) | 0.31 | -0.03 (-0.1, 0.03) | 0.33 | 0.01 (-0.06, 0.08) | 0.79 |
|  |  |  |  |  |  |  |  |  |  |
| Adjusting for gestational age at sample collection | PFOA | 0.09 (0.01, 0.18) | 0.03 | -0.02 (-0.1, 0.05) | 0.5 | -0.03 (-0.1, 0.05) | 0.49 | 0 (-0.08, 0.08) | 1 |
|  | PFOS | 0.08 (0, 0.15) | 0.06 | 0 (-0.06, 0.07) | 0.94 | 0.03 (-0.05, 0.1) | 0.48 | 0.03 (-0.04, 0.11) | 0.38 |
|  | PFNA | 0.04 (-0.02, 0.11) | 0.2 | 0 (-0.05, 0.05) | 0.99 | -0.04 (-0.1, 0.02) | 0.17 | -0.01 (-0.08, 0.05) | 0.67 |
|  | PFHxS | 0.05 (-0.01, 0.11) | 0.13 | -0.01 (-0.07, 0.04) | 0.68 | 0.01 (-0.05, 0.08) | 0.67 | 0 (-0.06, 0.07) | 0.88 |
|  | PFDA | 0.04 (-0.02, 0.11) | 0.2 | 0.02 (-0.03, 0.07) | 0.54 | -0.01 (-0.07, 0.05) | 0.81 | 0.02 (-0.05, 0.08) | 0.6 |
|  | PFUA | 0.05 (-0.02, 0.12) | 0.18 | 0.02 (-0.04, 0.09) | 0.48 | -0.04 (-0.11, 0.02) | 0.2 | 0 (-0.07, 0.07) | 0.97 |
|  |  |  |  |  |  |  |  |  |  |
| Excluding absolute values of ultrasound Z-scores above 4 | PFOA | 0.1 (0.01, 0.18) | 0.02 | -0.02 (-0.09, 0.06) | 0.66 | -0.02 (-0.1, 0.05) | 0.58 | 0.01 (-0.07, 0.09) | 0.86 |
|  | PFOS | 0.07 (-0.01, 0.15) | 0.07 | 0 (-0.07, 0.07) | 0.98 | 0.02 (-0.05, 0.1) | 0.51 | 0.03 (-0.04, 0.11) | 0.4 |
|  | PFNA | 0.04 (-0.02, 0.11) | 0.18 | 0.01 (-0.05, 0.06) | 0.85 | -0.04 (-0.1, 0.02) | 0.19 | -0.01 (-0.07, 0.05) | 0.76 |
|  | PFHxS | 0.06 (-0.01, 0.12) | 0.07 | -0.01 (-0.06, 0.05) | 0.84 | 0.02 (-0.04, 0.08) | 0.58 | 0.01 (-0.05, 0.08) | 0.71 |
|  | PFDA | 0.04 (-0.02, 0.11) | 0.19 | 0.02 (-0.03, 0.07) | 0.48 | -0.01 (-0.07, 0.05) | 0.8 | 0.02 (-0.05, 0.08) | 0.58 |
|  | PFUA | 0.04 (-0.03, 0.12) | 0.22 | 0.02 (-0.04, 0.09) | 0.51 | -0.04 (-0.11, 0.02) | 0.19 | 0 (-0.07, 0.07) | 0.93 |
| Note: Estimates derived from generalized estimating equations with exchangeable correlation. Effect estimates (95% confidence intervals) show the change in standard deviations (or raw values measured in mm for FL, HC, and AC, and gram for EFW) measure per interquartile range increase in log-transformed PFAS concentrations. Models adjusted for maternal age, prepregnancy BMI, race/ethnicity, education, insurance status, parity, enrollment year, fetal sex and number of ultrasound scans. While running models with raw values, a nonlinear spline term for gestational age at ultrasound was added as a covariate. The third sensitivity analysis additionally adjusted for gestational age at PFAS measurement (weeks). The sample size (number of records, participants) for each analysis was: 1. Excluding smokers [FL (4641, 1092); HC (4488, 1045); AC (4514, 1045); EFW (4509, 1045)] 2. Adjusting for GA [FL (4980, 1173); HC (4825, 1126); AC (4854, 1126); EFW (4847, 1126)] 3. Excluding absolute Z-scores > 4 [FL (4970, 1173); HC (4816, 1126); AC (4845, 1126); EFW (4838, 1126)]. While running models excluding smokers, we excluded all participants with missing values for smoking and those who reported smoking. | | | | | | | | | |

Table S12. Associations between prenatal PFAS exposure and birthweight (or Z-scores) among all participants and across different subsets.

| PFAS | Sensitivity analysis | N | Estimate (95% CI) | p-value |
| --- | --- | --- | --- | --- |
| PFOA | Excluding smokers | 1087 | 0.02(-0.08, 0.13) | 0.679 |
|  | Adjusting for gestational age at sample collection | 1168 | -0.01(-0.11, 0.09) | 0.839 |
|  | Excluding absolute values of ultrasound Z-scores above 4 | 1164 | 0(-0.1, 0.1) | 0.979 |
|  | Using raw birthweight | 1168 | -0.36(-43.96, 43.25) | 0.987 |
|  |  |  |  |  |
| PFOS | Excluding smokers | 1087 | 0.05(-0.05, 0.15) | 0.327 |
|  | Adjusting for gestational age at sample collection | 1168 | 0.03(-0.07, 0.13) | 0.526 |
|  | Excluding absolute values of ultrasound Z-scores above 4 | 1164 | 0.03(-0.07, 0.12) | 0.577 |
|  | Using raw birthweight | 1168 | 21.58(-20.64, 63.8) | 0.317 |
|  |  |  |  |  |
| PFNA | Excluding smokers | 1087 | 0.01(-0.07, 0.1) | 0.742 |
|  | Adjusting for gestational age at sample collection | 1168 | 0(-0.08, 0.08) | 0.985 |
|  | Excluding absolute values of ultrasound Z-scores above 4 | 1164 | 0(-0.08, 0.08) | 0.927 |
|  | Using raw birthweight | 1168 | 4.68(-30.4, 39.76) | 0.794 |
|  |  |  |  |  |
| PFHxS | Excluding smokers | 1087 | 0(-0.08, 0.09) | 0.972 |
|  | Adjusting for gestational age at sample collection | 1168 | -0.01(-0.1, 0.07) | 0.745 |
|  | Excluding absolute values of ultrasound Z-scores above 4 | 1164 | -0.02(-0.1, 0.06) | 0.694 |
|  | Using raw birthweight | 1168 | -1.66(-36.91, 33.59) | 0.926 |
|  |  |  |  |  |
| PFDA | Excluding smokers | 1087 | 0.05(-0.03, 0.12) | 0.239 |
|  | Adjusting for gestational age at sample collection | 1168 | 0.04(-0.04, 0.11) | 0.314 |
|  | Excluding absolute values of ultrasound Z-scores above 4 | 1164 | 0.03(-0.04, 0.11) | 0.366 |
|  | Using raw birthweight | 1168 | 18.46(-13.18, 50.09) | 0.253 |
|  |  |  |  |  |
| PFUA | Excluding smokers | 1087 | 0.04(-0.06, 0.14) | 0.409 |
|  | Adjusting for gestational age at sample collection | 1168 | 0.01(-0.08, 0.11) | 0.752 |
|  | Excluding absolute values of ultrasound Z-scores above 4 | 1164 | 0(-0.09, 0.09) | 0.97 |
|  | Using raw birthweight | 1168 | 5.33(-33.72, 44.38) | 0.789 |
| Note: Associations between prenatal PFAS exposure and standardized birthweight measurements estimated using linear regression models. Coefficients (95% CI) represent changes in birthweight (or birthweight Z-score) per interquartile range increase in log‐transformed PFAS concentrations. All models were adjusted for maternal age, prepregnancy BMI, race/ethnicity, education, insurance status, parity, enrollment year, and fetal sex. Models for birthweight raw scores (gram) were additionally adjusted for gestational age at delivery. | | | | |

Table S13. Model fit statistics for latent class trajectory models (N = 1220).

| No. of classes | Polynomial order | BIC | AIC | % in each class | Average posterior probability | Entropy |
| --- | --- | --- | --- | --- | --- | --- |
| 3^1^ | HC: 3, 3, 3; AC: 3, 3, 3; FL: 3, 3, 3 | -11147.35 | -11043.55 | 21.4 (group 1), 53.0 (group 2), 25.6 (group 3) | 0.91 | 0.81 |
| 4^1^ | HC: 3, 3, 3, 3; AC: 3, 3, 3, 3; FL: 3, 3, 3, 3 | -11073.68 | -10936.97 | 9.6 (group 1), 35.3 (group 2), 40.1 (group 3), 15.0 (group 4) | 0.87 | 0.78 |
| 5^1^ | HC: 3, 3, 3, 3, 3; AC: 3, 3, 3, 3, 3; FL: 3, 3, 3, 3, 3 | -11029.76 | -10860.14 | 8.1 (group 1), 22.8 (group 2), 34.7 (group 3), 20.5 (group 4), 13.9 (group 5) | 0.82 | 0.74 |
| 4^2^ | HC: 1, 1, 2, 1; AC: 3, 1, 2, 3; FL: 3, 1, 3, 2 | -11035.05 | -10931.26 | 9.6 (group 1), 34.9 (group 2), 40.4 (group 3), 15.2 (group 4) | 0.87 | 0.77 |
| Note: ^1^ Model fit statistics for 3-5 class solutions using cubic polynomial trajectories for each growth parameter. ^2^ Final selected 4-class model after refining polynomial orders to retain only significant or linear terms. | | | | | | |
